# Differences in the skeletal muscle transcriptome profile associated with extreme values of fatty acids content

**DOI:** 10.1186/s12864-016-3306-x

**Published:** 2016-11-22

**Authors:** Aline S. M. Cesar, Luciana C. A. Regitano, Mirele D. Poleti, Sónia C. S. Andrade, Polyana C. Tizioto, Priscila S. N. Oliveira, Andrezza M. Felício, Michele L. do Nascimento, Amália S. Chaves, Dante P. D. Lanna, Rymer R. Tullio, Renata T. Nassu, James E. Koltes, Eric Fritz-Waters, Gerson B. Mourão, Adhemar Zerlotini-Neto, James M. Reecy, Luiz L Coutinho

**Affiliations:** 1 Department of Animal Science, University of São Paulo, Piracicaba, SP 13418-900 Brazil; 2 Department of Animal Science, Iowa State University, Ames, IA 50011 USA; 3 Embrapa Pecuária Sudeste, São Carlos, SP 13560-970 Brazil; 4 Departament of Genetics and Evolutionary Biology-IB, USP, São Paulo, SP 05508-090 Brazil; 5 Department of Animal Science, University of Arkansas, Fayetteville, AR 72701 USA; 6 Embrapa Informática Agropecuária, Campinas, SP 13083-886 Brazil

**Keywords:** Lipids, RNA-Seq, *Bos indicus*, Global Oxidative Metabolism, Human Health

## Abstract

**Background:**

Lipids are a class of molecules that play an important role in cellular structure and metabolism in all cell types. In the last few decades, it has been reported that long-chain fatty acids (FAs) are involved in several biological functions from transcriptional regulation to physiological processes. Several fatty acids have been both positively and negatively implicated in different biological processes in skeletal muscle and other tissues. To gain insight into biological processes associated with fatty acid content in skeletal muscle, the aim of the present study was to identify differentially expressed genes (DEGs) and functional pathways related to gene expression regulation associated with FA content in cattle.

**Results:**

Skeletal muscle transcriptome analysis of 164 Nellore steers revealed no differentially expressed genes (DEGs, FDR 10%) for samples with extreme values for linoleic acid (LA) or stearic acid (SA), and only a few DEGs for eicosapentaenoic acid (EPA, 5 DEGs), docosahexaenoic acid (DHA, 4 DEGs) and palmitic acid (PA, 123 DEGs), while large numbers of DEGs were associated with oleic acid (OA, 1134 DEGs) and conjugated linoleic acid cis9 trans11 (CLA-c9t11, 872 DEGs). Functional annotation and functional enrichment from OA DEGs identified important genes, canonical pathways and upstream regulators such as *SCD*, *PLIN5*, *UCP3*, *CPT1*, *CPT1B*, oxidative phosphorylation mitochondrial dysfunction, *PPARGC1A,* and *FOXO1*. Two important genes associated with lipid metabolism, gene expression and cancer were identified as DEGs between animals with high and low CLA-c9t11, specifically, epidermal growth factor receptor (*EGFR)* and *RNPS*.

**Conclusion:**

Only two out of seven classes of molecules of FA studied were associated with large changes in the expression profile of skeletal muscle. OA and CLA-c9t11 content had significant effects on the expression level of genes related to important biological processes associated with oxidative phosphorylation, and cell growth, survival, and migration. These results contribute to our understanding of how some FAs modulate metabolism and may have protective health function.

**Electronic supplementary material:**

The online version of this article (doi:10.1186/s12864-016-3306-x) contains supplementary material, which is available to authorized users.

## Background

Lipids are a class of molecules present in all cell types. They contribute to cellular structure, energy storage and several biological functions from transcriptional regulation to physiological processes [1]. Over the last couple decades, it has been reported that consumption of fatty acids is associated with fat deposition and may have metabolic effects, such as altered blood lipid and lipoprotein content [2]. However, there are still contrasting opinions about the role of dietary fatty acids on human health, which can be observed in previous reviews and meta-analysis studies conducted in the last years [3, 4].

Lipid composition in beef has become an important discussion point. Although meat is a significant source of fat in the human diet, it contains high saturated fat content that is associated with some diseases such as obesity, cancer and coronary heart disease [5, 6]. On the other hand, meat has high nutritional value and is an important source of unsaturated fatty acids such as oleic acid (OA) and conjugated linoleic acid (CLA), which have beneficial effects on human health [7]. Thus, there is a need to improve the nutritional value of meat and better understand the biological and molecular processes associated with fatty acid composition in skeletal muscle.

Previous studies have shown that OA as well as other fatty acids such as linoleic acid (LA), docosahexaenoic acid (DHA), eicosapentaenoic acid (EPA) and CLA can regulate gene transcription in tissues such as muscle, liver, adipose, monocytes and blood mononuclear cells [8, 9]. Activation of nuclear receptors such as peroxisome proliferator-activated receptors (*PPARs*) and coactivators of the sterol response element binding protein (*SREBP*) can mediate this regulation. Both *PPARs* and *SREBPs* play a role in preadipocyte differentiation, lipid homeostasis and peroxisomal beta-oxidation regulation by controlling the transcription of acyl-CoA oxidase and other enzymes involved in adipocyte development [10–12]. While elevated intracellular levels of stearic (SA) and palmitic (PA) acids were associated with apoptotic death [13] and inflammatory process [14]. However, the effective involvement of these fatty acids in molecular and physiological processes in skeletal muscle remains unclear.

The present investigation was undertaken to identify differentially expressed genes (DEGs) and functional pathways associated with seven FA content in beef cattle. Our hypothesis was that variation in FA content could be associated with difference in gene expression in skeletal muscle. Our study revealed that up regulation of stearoyl-CoA desaturase (*SCD*) is associated with deposition of unsaturated FA and that increased levels of OA and CLA are associated with expression of genes that have protective function associated with important human diseases.

## Results

### Phenotypes and sequencing data

Seven different FAs, important to beef quality and human health, were used in this transcriptome study, including: oleic acid (OA, C18:1 cis9), palmitic acid (PA, 16:0), stearic acid (SA, C18:0), linoleic acid (LA, C18:2 cis9,12), conjugated linoleic acid cis9 trans11 (CLA-c9t11, C18:2 cis9 trans11), eicosapentaenoic acid (EPA, C22:5) and docosahexaenoic acid (DHA, C22:6). The skeletal muscle samples analyzed were chosen based on their extreme values for each FA studied (Additional file 1). When ranked by fatty acids content in skeletal muscle, the statistical test of means performed between the high (H) and low (L) groups according to FA had significant differences (*p*-value < 0.05) for OA, PA, SA, CLA-c9t11, LA, EPA and DHA, but no difference in IMF content or backfat thickness (Additional file 2). However, animals with extreme phenotypes for OA, PA, SA, CLA-c9t11, LA, EPA and DHA also had significant differences for other FAs. For example, animals with high levels of OA had significantly (*p* < 0.05) lower levels for SA and PA, but high levels for CLA-c9t11. While extreme animals for CLA-c9t11 had significant low levels for SA and PA, but high levels for OA and DHA. The correlation between any two FAs tended to be low (<0.2). However, there were some FAs that high negative correlations, e.g. OA and PA (-0.63) and OA and SA (-0.62) or high positive correlations, e.g. EPA and LA (0.66) and EPA and DHA (0.74) (Table 1).

**Table 1:** Phenotypic correlation between the groups of extreme values of fatty acids content in skeletal muscle

Fatty acids	PA^a^	SA^b^	OA^c^	LA^d^	CLA-c9t11^e^	EPA^f^	DHA^g^
PA	1	0.16	−0.63	−0.02	−0.15	−0.15	−0.15
SA	0.16	1	−0.62	0.02	−0.31	−0.02	0.05
OA	−0.63	−0.62	1	0.06	0.51	0.06	0.08
LA	−0.02	0.02	0.06	1	0.05	0.66	0.53
CLA-c9t11	−0.15	−0.31	0.51	0.05	1	0.07	0.17
EPA	−0.15	−0.02	0.06	0.66	0.07	1	0.74
DHA	−0.15	0.05	0.08	0.53	0.17	0.74	1

^a^Palmitic acid

^b^Stearic acid

^c^Oleic acid

^d^Linoleic acid

^e^Conjugated linoleic acid cis9, trans 11

^f^Eicosapentaenoic acid

^g^Docosahexaenoic acid

The average number of mapped read pairs per sample before and after filtering were 14.47 million and 11.5 million respectively (see Additional file 3), which means that on average 75.85% of total read pairs were mapped to the *Bos taurus* UMD3.1 reference genome assembly. After filtering for transcript expression level, a total of 17,137; 17,070; 17,053; 17,052; 17,038; 16,989 and 17,100 annotated genes were used for differential expression analysis for OA, PA, SA, CLA-c9t11, EPA, DHA and LA content, respectively.

### Differential expression analysis

**Fig. 1:**
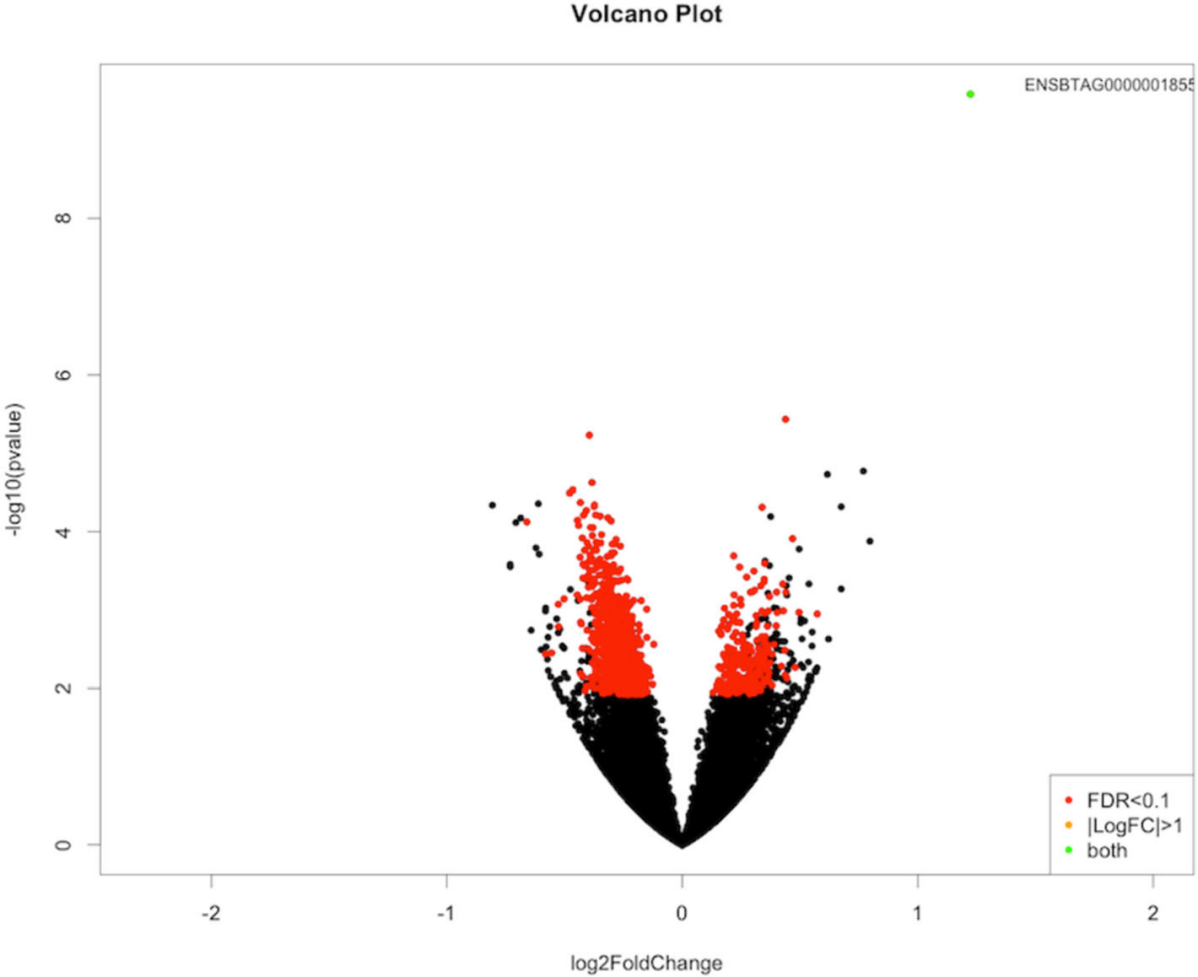
Volcano plot of log2fold-change (x-axis) versus − log10FDR-corrected *p*-value in RNA-Seq data for oleic acid content

Differential gene expression analysis was performed for each FA by camparing gene expression level between the H and L FA content groups. No DEGs were identified for LA and SA (see Additional files 4 and 5), while 5, 4 and 123 DEGs were identified for EPA, DHA and PA, respectively (see Additional files 6, 7 and 8). In contrast, 1134 and 872 DEGs for OA and CLA-c9t11, respectively, were identified between H and L groups (for more details see Additional file 9 and 10). A Volcano plot of log2 fold-change (x-axis) versus − log10 FDR-corrected *p*-value (y-axis) for OA is shown in Fig. 1. The volcano plot of other FAs are shown in Additional file 11. Of the 1134 DEGs for OA, 870 genes were up-regulated and 264 were down-regulated and of the 872 DEGs for CLA-c9t11, 536 genes were up-regulated and 336 were down-regulated in the L group relative to the H group. The expression level, fold change, adjusted *p*-value and annotation of all DEGs for OA and CLA-c9t11 are shown in more detail in Additional files 9 and 10.

### Functional enrichment analysis for oleic acid content

In this study, two different tools of pathway analysis were performed: DAVID and Ingenuity Pathway Analysis® (IPA®). According to DAVID enrichment analysis, several ontology terms were significantly enriched (*p*-value adjusted for multiple testing by Benjamin-Hochberg [15], BH-adj-*p*value) for the differentially expressed gene lists (see Additional file 12). Cellular component GO terms related to mitochondrial envelope (BH-adj-*p*value = 2.6e-15), mitochondrial membrane (BH-adj-*p*value = 1.0e-14), respiratory chain (BH-adj-*p*value = 6.6e-12), mitochondrial matrix (BH-adj-*p*value = 6.0e-08) and intracellular organelle lumen (BH-adj-*p*value = 5.8e-06) were associated with DEGs for OA content. In addition, six KEGG pathways (BH-adj-*p*-value < 0.10) that were associated with oxidative phosphorylation (BH-adj-*p*value = 5.8e-10), ribosome (BH-adj-*p*value = 1.6e-02) and proteasome (BH-adj-*p*value = 2.9e-02) were also identified. These pathways are involved with oxidative phosphorylation and oxidative stress diseases such as Huntington (BH-adj-*p*value = 3.0E-10), Parkinson (BH-adj-*p*value = 1.5e-08) and Alzheimer (BH-adj-*p*value = 1.2E-07) (Table 2).

**Table 2:** Pathways identified from differentially expressed genes list between high and low oleic acid content

Category	Term	Count^a^	%^b^	*P*-Value	BH-adj^c^
KEGG_PATHWAY	Oxidative phosphorylation	33	3.0	3.7e-12	5.8e-10
KEGG_PATHWAY	Huntington’s disease	38	3.5	3.9e-12	3.0e-10
KEGG_PATHWAY	Parkinson’s disease	30	2.8	2.9e-10	1.5e-8
KEGG_PATHWAY	Alzheimer’s disease	32	3.0	3.2e-9	1.2e-7
KEGG_PATHWAY	Ribosome	15	1.4	5.0e-4	1.6e-2
KEGG_PATHWAY	Proteasome	10	0.9	1.1e-3	2.9e-2

^a^Number of differentially expressed genes involved in the term

^b^Percentage of differentially expressed genes involved in the term

^c^ *P*-value adjusted for multiple tests by Benjamin and Hochberg (1995)

The IPA® software (IPA®, QIAGEN Redwood City, www.qiagen.com/ingenuity) was also performed for functional enrichment using the Global Functional Analysis (GFA) and Global Canonical Pathways (GCP) analysis from the DEGs list for OA content (1134 genes). Global Canonical Pathways analysis estimates the likelihood that the association between a set of DEGs in a pathway is due to random chance using a right-tailed Fishers Exact Test. Significance was set at BH-adj-*p*value ≤ 0.05, which indicated a non-random association. Several canonical pathways (BH-adj-*p*value ≤ 0.05, which indicated a non-random association) were identified by GCP analysis, including oxidative phosphorylation (BH-adj-*p*value = 7.94e-17), mitochondrial dysfunction (BH-adj-*p*value = 3.98e-16), insulin receptor signaling (BH-adj-pvalue = 0.02), *LPS*-stimulated MAPK signaling (BH-adj-*p*value = 0.035), role of NFAT in cardiac hypertrophy (BH-adj-*p*value = 0.035) and Huntington’s disease signaling (BH-adj-*p*value = 0.04) (see Additional file 13).

**Fig. 2:**
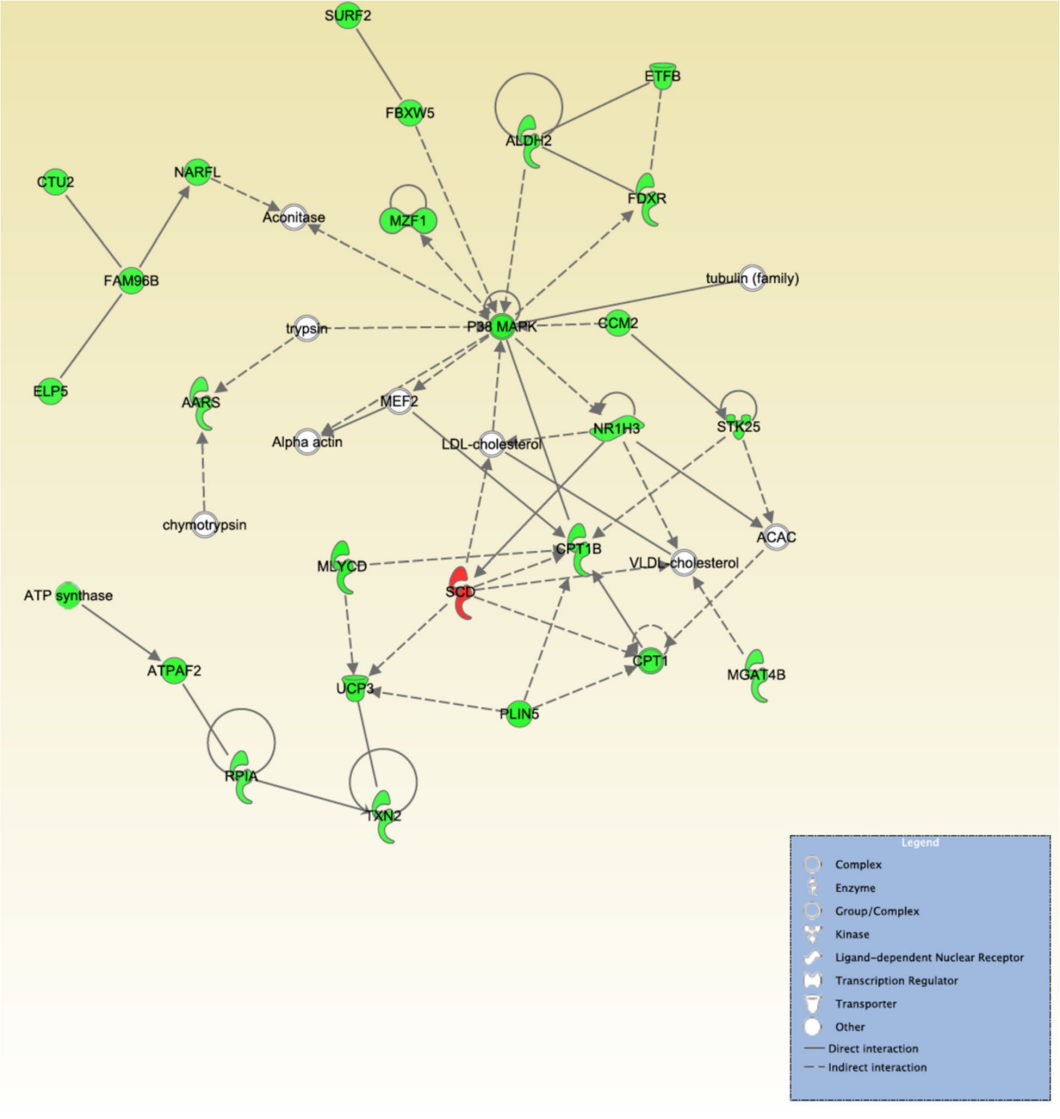
A gene network associated with lipid metabolism and energy production is impacted by OA content. Genes presented in *red* are up-regulated in the high oleic acid content (H) group. Genes presented in *green* are down-regulated in the H group. The intensity of the colors is related to the estimated of fold change. Molecules in *white* are not in the DE list, but were incorporated into the network through relationships with other molecules

Twenty-five networks were enriched as a function of OA content as identified by IPA®GFA analyses (see Additional file 14). One in particular, shown in Fig. 2, was associated with lipid metabolism and energy production. This network contained molecules and genes such as cholesterol (LDL-cholesterol), very-low-density lipoprotein (VLDL-cholesterol), perilipin 5 (*PLIN5*), *SCD*, low-density lipoprotein carnitine palmitoyltransferase I (*CPT1*) and carnitine palmitoyltransferase I (*CPT1B*), respectively (Fig. 2). Among these only *SCD* was up-regulated in the H group as shown in red in Fig. 2.

**Fig. 3:**
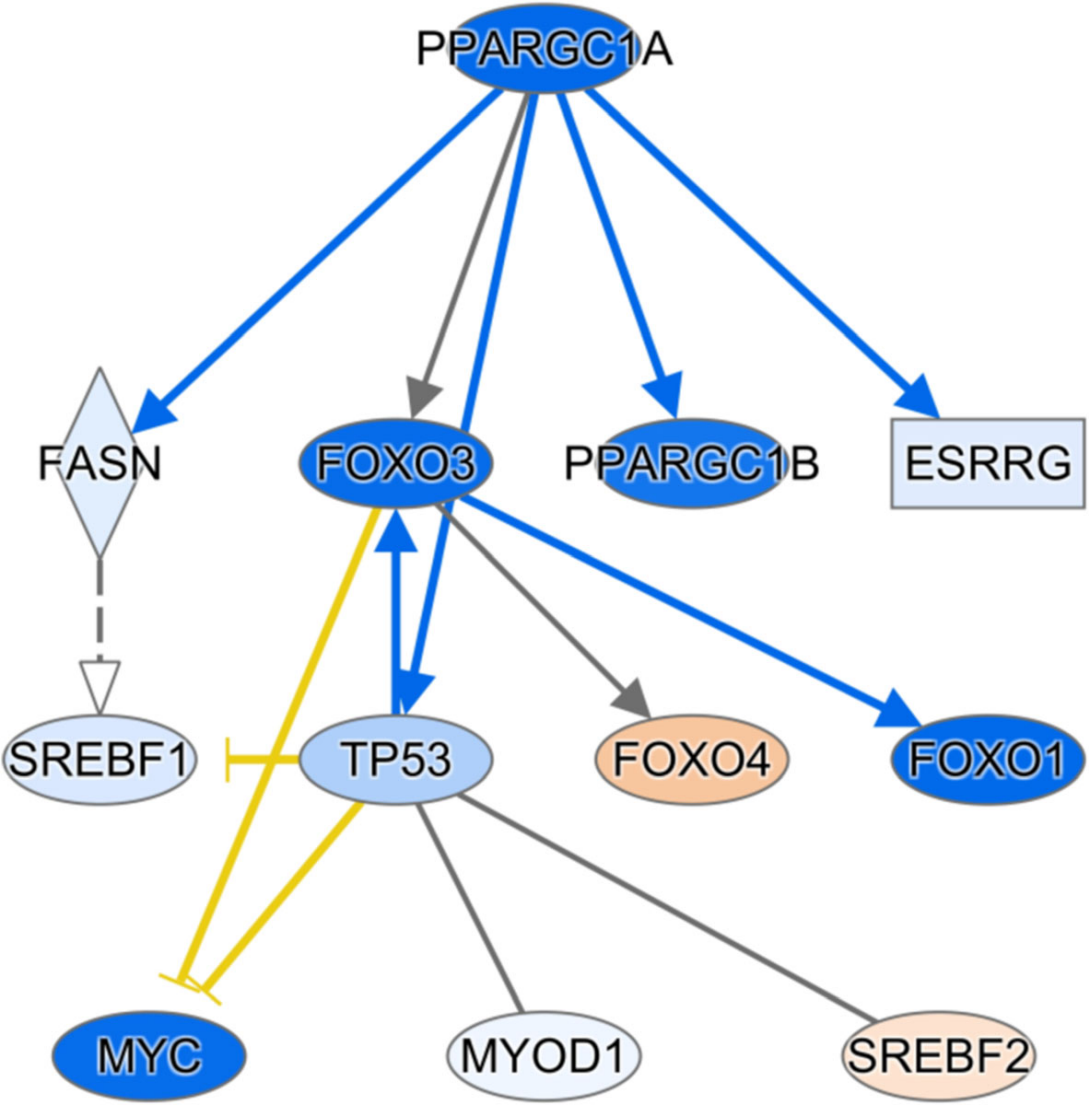
The mechanistic network of the upstream regulators and their relationship predicted by IPA®. The molecules shown in *blue* are predicted to be inhibited, while the molecules shown in *orange* are predicted to be activated as a result of OA content. Color intensity represents the level of inhibition or activation. The *lines* and *arrows* in *blue* represent a direct interaction and activation, respectively. The interrupted lines in *yellow* represents direct inhibition. The *gray* lines represent the interactions that OA content did not affect, but have been reported in the literature

To further explore the observed changes in gene expression, IPA® upstream regulator analyses were performed to identify the cascade of upstream transcriptional regulators that could be involved in the gene expression changes in skeletal muscle due to OA content. The IPA® program estimated the effects between transcriptional regulators and their target genes based on prior knowledge stored in the Ingenuity® Knowledge Base. Upstream regulators were identified from the list of DEGs for OA content (see Additional file 15), which were connected via mechanistic networks detected by IPA®. Peroxisome proliferator-activated receptor gamma, coactivator 1 alpha (*PPARGC1A*) was among the top predicted upstream regulators. *PPARGC1A* and ten other transcription factors (TFs) such as forkhead box O3 (*FOXO3*), peroxisome proliferator-activated receptor gamma, coactivator 1 beta (*PPARGC1B*), estrogen-related receptor gamma (*ESRRG*), sterol regulatory element binding transcription factor 1 (*SREBF1*), tumor protein p53 (*TP53*), forkhead box O1 (*FOXO1*), V-Myc avian myelocytomatosis viral oncogene homolog (*MYC*) and myogenic differentiation 1 (*MYOD1*) were predicted to be inhibited (blue shapes in Fig. 3), whereas the transcription factors forkhead Box O4 (*FOXO4*) and sterol regulatory element binding transcription factor 2 (*SREBF2*) were predicted to be activated (orange shapes in Fig. 3).

**Fig. 4:**
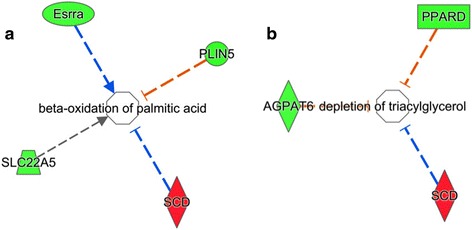
Biological functions that include stearoyl-CoA desaturase such as (**a**) beta-oxidation of palmitic acid and (**b**) AGPAT6 depletion of triacylglycerol. Genes shown in *red* are up-regulated in the high oleic acid content group, while genes shown in *green* are down-regulated

The functional enrichment analysis using the IPA® program identified several statically significantly enriched biological functions (BH-adj-*p*-value ≤ 0.05) from the OA DEGs list (see Additional file 16), e.g. processing of RNA (BH-adj-*p*value = 2.28e-07, mitochondrial disorder (BH-adj-*p*value = 3.46e-07), transcription of RNA (BH-adj-*p*value = 1.70e-06), expression of RNA (BH-adj-*p*value = 3.30e-06), transcription of DNA (BH-adj-*p*value = 2.51e-05), expression of mRNA (BH-adj-*p*value = 4.96e-05), catabolism of fatty acid (BH-adj-*p*value = 1.86e-03), beta-oxidation of palmitic acid (BH-adj-*p*value = 3.20e-03), depletion of triacylglycerol (BH-adj-*p*value = 9.34e-04 and oxidation of long chain fatty acid (BH-adj-*p*value = 2.86e-02). The indirect inhibitory interaction between *SCD* and β-oxidation and the depletion of triacylglycerol, which was associated with lipid metabolism and energy production are shown in Fig. 4. In the network shown in Fig. 2 it is also possible to observe the indirect interaction between *SCD*, LDL-cholesterol, VLDL-cholesterol, *CPT1*, *CPT1B*, *PLIN5* and *UCP3*.

### Functional enrichment analysis for conjugated linoleic acid cis9 trans11 content

The enrichment analysis performed by DAVID software using the list of DEGs for CLA-c9t11 content identified one KEGG pathway, which was related to ribosome function (BH-adj-*p*value = 1.2e-02). Among the molecular functions identified by DAVID associated (BH-adj-*p*value < 0.05) with the CLA-c9t11 DE genes identified between the groups with extreme values of CLA-c9t11 were nucleotide binding (BH-adj-*p*value = 1.7e-03), ATP binding (BH-adj-*p*value = 2.2e-02) and structural constituent of ribosome (BH-adj-*p*value = 2.4e-02) (see Additional file 17).

The top five canonical pathways identified for CLA-c9t11 content by GFA and GCP analysis using IPA® program were protein ubiquitination (nominal *p*value = 4.11e-07), interferon signaling (nominal *p*value = 2.9e-05), hypoxia signaling in the cardiovascular system (nominal *p*value = 8.8e-05), angiopoietin signaling (nominal *p*value = 1.01e-04) and mTOR signaling (nominal *p*value = 1.19e-04) (see Additional file 18).

**Fig. 5:**
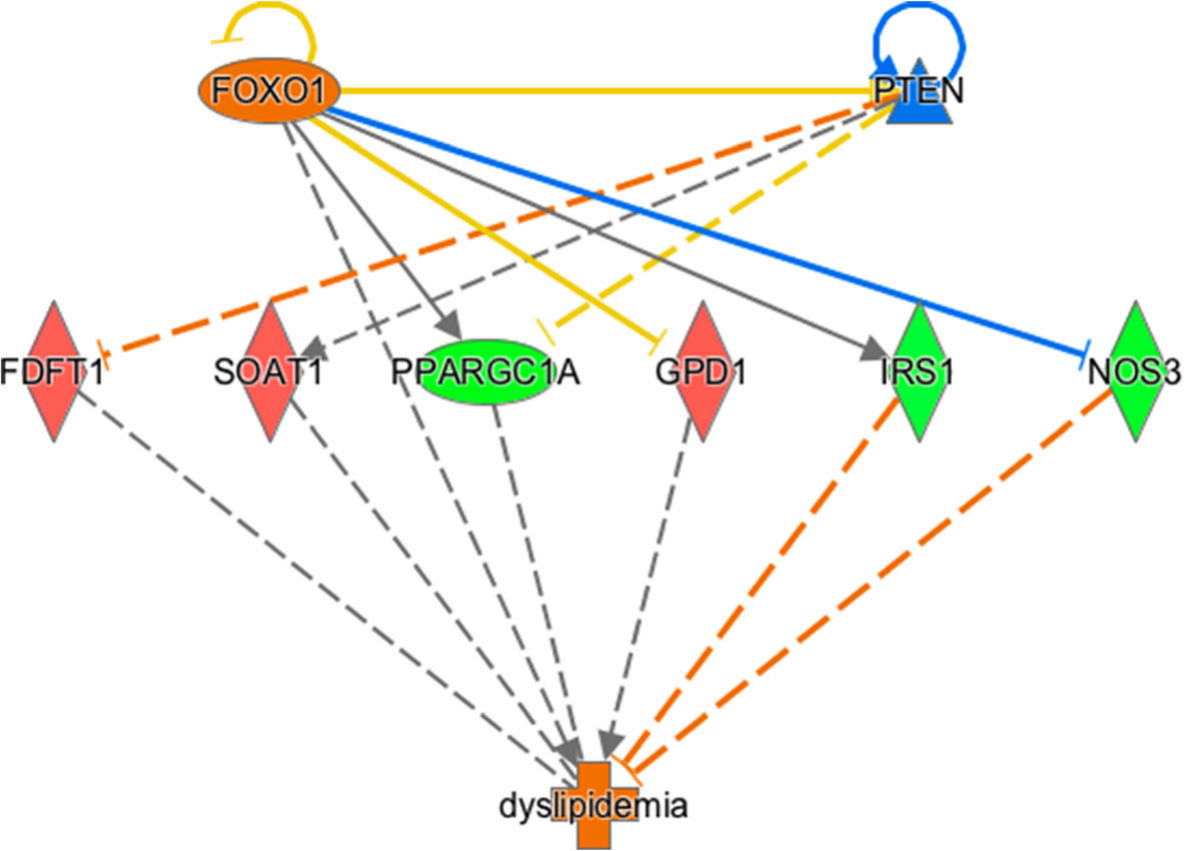
The network of the upstream regulator *FOXO1* by IPA®. Genes presented in *red* are up-regulated in the high oleic acid content (H) group. Genes presented in *green* are down-regulated in the H group. The intensity of the colors is related to the estimated of the fold change. The interrupted lines in *orange* represents direct inhibition. The *blue* represents direct activation. The *gray* lines represent the no interactions have been reported in the literature

The top five upstream regulators identified from the list of DEGs for CLA-c9t11, associated network functions and toxicology list (list of molecules/genes that are relevant to causality of the phenotype of interest, which can help to identify potential therapeutic or toxicity targets) for CLA-c9t11 content are shown in Additional file 18. Among the top upstream regulators were: interferon, lambda 1 (*IFLN1*), tumor necrosis factor (*TNF*), retinoic acid, signal transducer and activator of transcription 2 (*STAT2*), and interferon regulatory factor 7 (*IRF7*), which were predicted as activated in this study (see Additional file 19). However other important cytokines and transcription regulators were identified as activated/inhibited such as *IFNK* (interferon kappa), *CCL5* (chemokine (C-C motif) ligand 5) and, *PRL* (prolactin), *TRIM24* (tripartite motif containing 24), *NFKB1A* (Nuclear factor NF-kappa-B 1A), *SREBF1*, *FOXO1, TP53* and *MYC*, respectively (see complete list in Additional file 19). Figure 5 shows the *FOXO1* network, which has been associated with the metabolic disorder dyslipidemia.

**Fig. 6:**
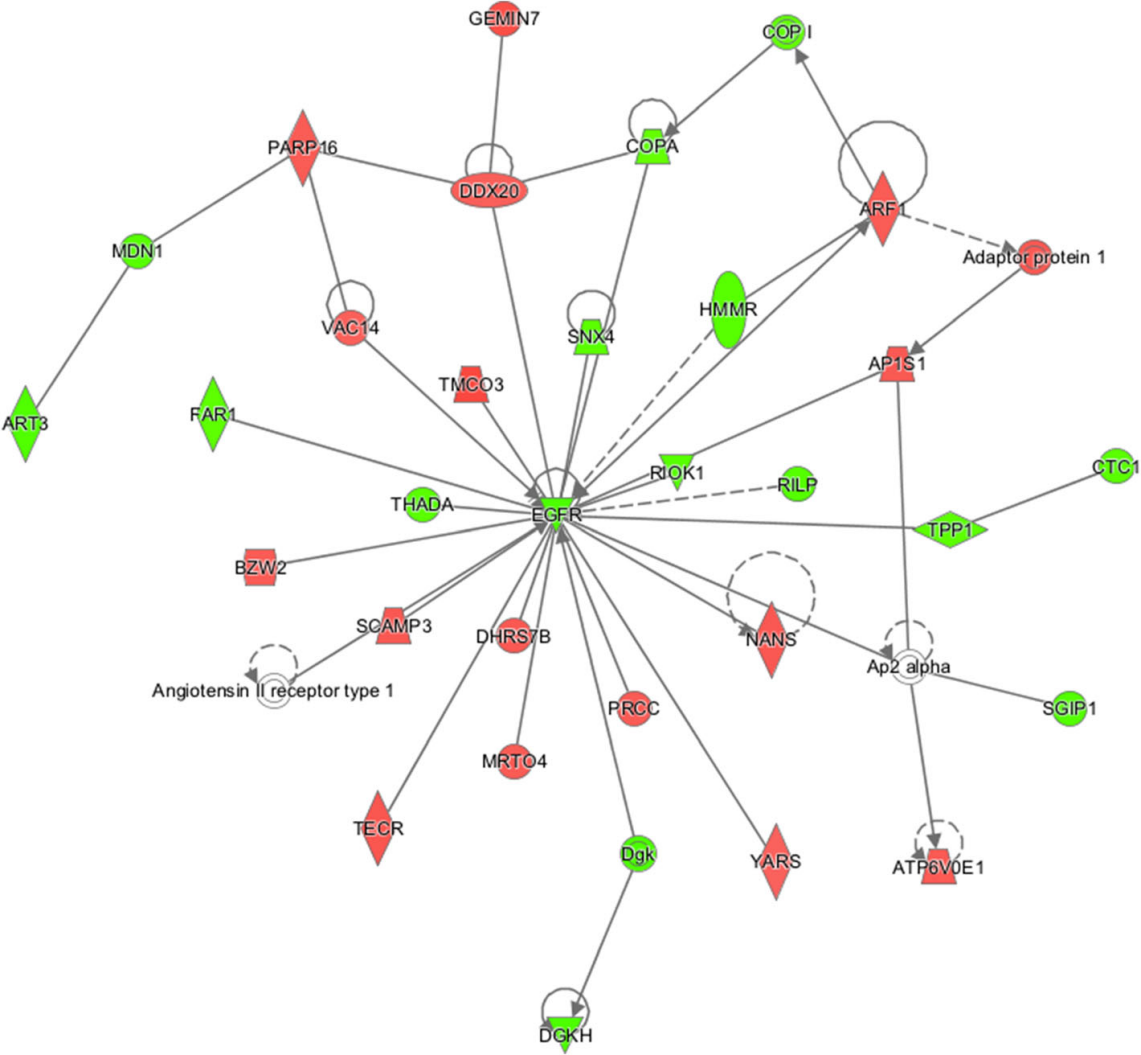
Network associated with lipid metabolism, nucleic-acid metabolism and small molecule biochemistry impacted by CLA-c9t11 content. Genes presented in *red* are up-regulated in the high CLA-c9t11 content (H) group. Genes presented in *green* are down-regulated in the H group. The intensity of the colors is related to estimate of the fold change. Molecules in *white* are not in the DE list, but were incorporated into the network through relationships with other molecules

**Fig. 7:**
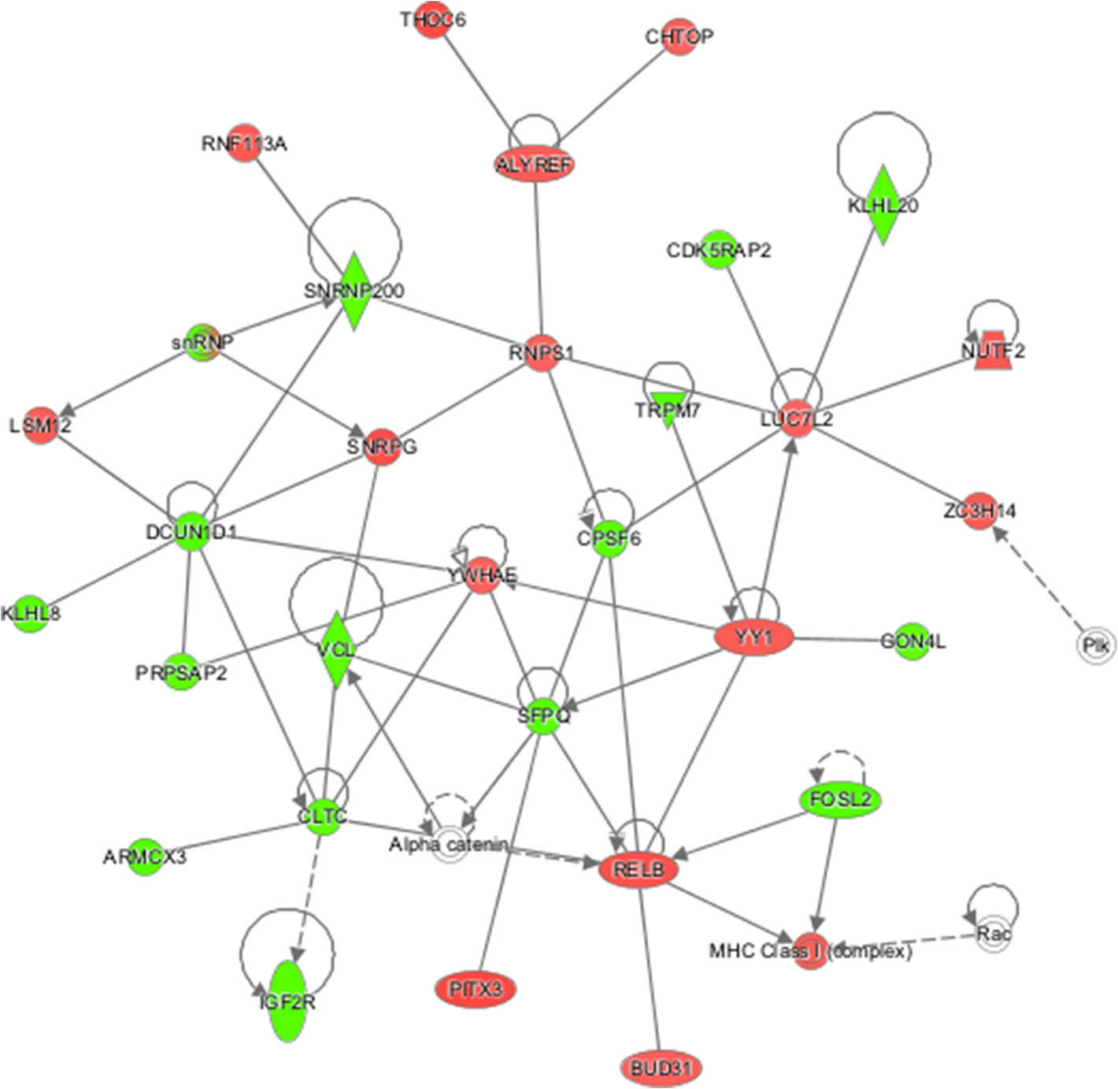
Network associated with molecular transport, RNA trafficking and RNA post-transcriptional modification impacted by CLA-c9t11 content. Genes presented in *red* are up-regulated in the high CLA-c9t11 content (H) group. Genes presented in *green* are down-regulated in the H group. The intensity of the colors is related to estimate of the fold change. Molecules in *white* are not in the DE list, but were incorporated into the network through relationships with other molecules

The top five associated network functions detected from the list of CLA-c9t11 DEGs between the groups with extreme values of CLA-c9t11 were related to RNA damage, repair and post-transcriptional modification; developmental disorder; metabolic disorder; lipid metabolism, molecular transport, RNA trafficking and; cell death and survival (see Additional file 18). Figure 6 illustrates the network associated with lipid and nucleic acid metabolism and small molecule biochemistry, which presents epidermal growth factor receptor (*EGFR*) as a central gene and which was down-regulated in animals with high CLA-c9t11 content (colored in green). Figure 7 shows a network associated with molecular transport, RNA trafficking, RNA post-transcriptional modification that contained ribonucleoproteins (*RNPS*), which were up-regulated in animals with high CLA-c9t11 content. The top five Toxicology terms were associated with cardiac necrosis/cell death (nominal *p*-value = 1.18e-06), cardiac hypertrophy (nominal *p*-value = 2.4e-04), hypoxia-inducible factor signaling (nominal *p*-value = 3.2e-03), increases liver steatosis (nominal *p*-value = 3.3e-03) and, *PPARA*/*RXRA* activation (nominal *p*-value = 5.3e-03) (see Additional file 18).

### Validation of differentially expressed genes

The quantitative real-time polymerase chain reaction (qRT-PCR) results and the relative quantification of DEGs selected to validate the present RNA-Seq study are shown in Additional file 20. Nine genes differently expressed between high and low OA groups were tested by qPCR and seven genes (78%) confirmed the RNAseq data (*p* < 0.1), one gene confirmed but not at a significant level and one gene disagreed with RNAseq data (Additional file 21).

## Discussion

Skeletal muscle is important for energy homeostasis, as it is a site of energy storage and insulin-stimulated glucose. Metabolic diseases such as obesity and coronary disorders could be a consequence of insulin resistance, i.e. the inability of insulin to drive glucose into the skeletal muscle and other tissues as liver, which can be caused by excessive body fat deposition [16]. Fatty acids in skeletal muscle play an important role in the structural and functional processes that influence the fluidity and stability of membrane structures, which impacts membrane functions such as transport, cell signaling, responses to oxidative damage and apoptosis. Studies have reported that the fatty acid profile of skeletal muscle phospholipids and triacylglycerides can be influenced by the fatty acid composition of human and animal diets [17, 18]. Several saturated and unsaturated fatty acids have been implicated, both positively and negatively, in different biological processes in skeletal muscle and other tissues such as liver and cardiac tissue [1, 19, 20]. In contrast, meta-analysis studies reported no evidence that a human diet, which was rich in monounsaturated fatty acids (MUFA) and polyunsaturated fatty acids (PUFA) and depleted for saturated fatty acid (SFA), had beneficial effects on coronary heart disease [3, 4].

In this study, all animals were of the same breed, sex, similar age and fed the same diet. However, different levels of FA content were observed in skeletal muscle. This maybe due to genetic variation among the animals [21] given that the environmental variables were similar across all animals. Transcriptomic analyses (validated by q RT-PCR [see Additional file 20]) indicated that many genes were differentially expressed (FDR 10%) between animals with high and low OA and CLA-c9t11 content. While, only a few genes were differentially expressed among the extreme groups for PA, SA, EPA and DHA and none for LA and SA. Additionally, previous studies reported only a few genes were affected by different levels of intramuscular fat content [22] and tenderness [23] in this same population.

Because the samples used for OA and CLA-c9t11 analysis also differed significantly for other FAs (see Additional file 2), we cannot be certain that the observed DE genes for OA and CLA-c9t11 are associated solely with these FAs. Despite the use of some samples in the analysis of several FA extremes, the lists of differentially expressed genes, and identified pathways were very different. This may not be surprising given the generally low correlation between FAs (Table 1). Thus it is very likely that utilization of only a couple samples in several analyses does not have an negative impact on other analyses. Finally, these findings indicate the biological importance of OA and CLA-c9t11 for skeletal muscle physiology. However, a word of caution is necessary because, a direct relation between gene expression level and protein abundance was not establish [24]. These fatty acids may play an important role in transcription regulation and consequently in energy homeostasis and metabolic diseases such as obesity and diabetes as also presented by Ntambi [25].

The most abundant MUFA found in skeletal muscle fat is oleic acid (OA, C18:1 cis9) [26]. It is present in membrane phospholipids, triglycerides and cholesterol esters. Human OA consumption has been associated with low levels of low-density lipoprotein (LDL) and also with the potential increase of high-density lipoprotein (HDL) levels in the blood. Furthermore, OA is converted to oleoylethanolamide (OEA), a small molecule in the intestine, which is involved in appetite control [27]. Previous studies have reported an important contribution of OA intake to general human health, which could lead to a decrease in cholesterol levels, atherosclerosis risk and diabetes occurrence. Further, OA has protective effects against viral infection and cancer development [28–30]. Beef has a high unsaturated fatty acid profile, primarily OA [28]. It is considered an important source of OA in human nutrition, and health [31–33]. Another important aspect of the OA content in beef is its relationship to meat quality traits such as tenderness, flavor and shelf-life [30]. Thus, it is appealing to improve our knowledge about biological processes associated with OA content in skeletal muscle, which could both help to improve the nutritional value and quality of beef.

In mammals, the biosynthesis of the OA is dependent on the activity of stearoyl-CoA desaturase (*SCD*). This soluble enzyme catalyzes the introduction of a *cis*-double bond at the 9^th^ carbon position of stearic acid (18:0), to form the MUFA, oleic acid (18:1) [30]. *SCD* is also required for the synthesis of highly unsaturated essential fatty acids or PUFAs such as eicosopentaenoic (EPA, C22:5 n-3) and docosahexaenoic acids (DHA, C22:6 n-6); synthesized from a-linolenic acid (LA, C18:2 cis9,cis12), and arachidonic acid (synthesized from linoleic acid), which are dependent on the presence of oleic acid [1]. In ruminants, such as beef cattle, the concentration of OA in adipose tissue depends more on the concentration of stearic acid hydrogenated by ruminal microorganisms than from stearic acid desaturation by *SCD* in the tissues [10].

EPA and DHA are omega-3 (n-3) long-chain PUFA, which are dietary fats associated with health benefits. They are present in cell membranes and play a role in anti-inflammatory processes and in the viscosity of cell membranes [34]. EPA and DHA are also precursors of several metabolites that are potent lipid mediators, considered by many investigators to be beneficial in the prevention or treatment of several diseases such as cardiovascular, obesity and Alzheimer [35–37]. However, in this study both EPA and DHA did not appear to have much of an effect on skeletal muscle gene expression, which may mean that they do not have a biological effect on skeletal muscle. It is interesting to speculate that DEG analyses of other tissues such as liver and adipose may be able to identify tissues responsive to these fatty acids. In contrast, Conjugated linoleic acid cis9, trans11 has a large effect on gene expression. This FA is associated with human health, specifically related with diseases such as cancer, immune and inflammatory responses and obesity [38, 39] and is naturally present in food sources such as fats in milk and meat of ruminant animals [39]. This may indicate that skeletal muscle is a primary target of Conjugated linoleic acid cis9, trans11.

The enriched transcript clusters identified by DAVID based on biological process GO terms (see Additional files 16 and 18) from the list of DEGs were associated with electron transport chain, mRNA translation and generation of precursor metabolites and energy. FAs like OA and CLA-c9t11 are involved in both extracellular and intracellular activities, which are in a partially dissociated state at physiological pH with part of the molecules diffused into the cytosol and other part integrating the membrane structure. These FAs impact several molecular functions in the cell such as: (1) ion transport across membranes; (2) activate intracellular enzymes; (3) modulation of Na+/K + -ATPase activation, (4) modulation of hormone/receptor interactions; (5) gene transcription and; (6) mRNA translation [40–44].

OA can be obtained by either dietary consumption or de novo lipogenesis. *De novo* lipogenesis of OA can occur from both dietary carbohydrates (will be transformed to triacylglycerol in case of positive energy balance) and fat. The enzyme responsible for MUFA OA biosynthesis is *SCD* [45], which is considered as a key enzyme in *de novo* lipogenesis, a process that is associated with obesity, synthesis of cholesterol and apoptosis. In the present study, *SCD* was up-regulated in the group of animals with high OA content. This was expected as this gene function is linked to OA synthesis. Hulver et al. [46] suggested that elevated expression of *SCD1* in human skeletal muscle contributes to abnormal lipid metabolism and progression of obesity. However, in this study, the IMF content between the L and H group was not statistically different (*p*-value = 0.06), as the H group had an average IMF content of 3.1% and the L group had an IMF of 2.7%. In this study, the fatty acid synthase (*FAS*) and acetyl-CoA carboxylase, responsible for fatty acid metabolism, were not differentially expressed between the groups H an L for any FA studied, considering that the diet was the same for all animals. This may indicate: 1) the increased *SCD1* in this study was associated just with higher synthesis of OA and not with higher fat deposition; 2) a difference in how bovine and human skeletal muscles respond to OA content.

Schrauwen [47], Clapham et al. [48] reported that mice which overexpressed *UCP3* in skeletal muscle had lower body weights and increased AMPK activity, elevated metabolic rate, lower fasting plasma glucose and insulin levels compared to wild type mice. In the first instance *UCP3*, which was down-regulated in the group of animals with high OA content was associated with regulation of energy homeostasis, but according to Schrauwen et al. [47] *UCP3* played an important role in the protection of mitochondria against lipid-induced oxidative damage by ROS production. This protection was the result of better fatty acid exportation by mitochondria and less production of hydrogen peroxide (H_2_O_2_), which caused oxidative damage to proteins and phospholipids. *PLIN5*, which was down-regulated in the group of animals with high OA content, in turn is a protein associated with lipid droplet that maintains the balance between lipogenesis and lipolysis and regulates fatty acid oxidation in oxidative tissues. These proteins recruit mitochondria to the surface of lipid droplets, which regulates their homeostasis by storing fatty acids in the form of triglycerides and releasing fatty acids for fatty acid oxidation by mitochondria [49]. *CPT1*, which was up-regulated in the group of animals with high OA content and *CPT1B*, which was down-regulated in the group of animals with high OA content, are important for the beta-oxidation of long chain fatty acids, which are involved in the long chain fatty acids transport to the mitochondrial matrix and associated with type 2 diabetes and insulin resistance. The inhibition of *CPT1* decreases the ability of muscles to oxidize fatty acids due to decreased long chain fatty acids transport into muscle mitochondria that could in turn be responsible for fat accumulation in skeletal muscle [50]. In this study, animals with lower values of OA content presented higher expression levels (Figs. 1 and 4) of *UCP3*, *PLIN5*, *CPT1* and *CPT1B* and lower expression level of *SCD*, corroborating with these previous studies [48–51] where the expression level of these genes were associated with fatty acid metabolism, energy homeostasis and animal health.

Herein, networks associated with reactive oxygen species (ROS) generation such as oxidative phosphorylation and mitochondrial dysfunction were detected, results that corroborate previous studies that reported the role of OA [52] and fatty acids [39] in the control of lipid oxidation. Mitochondria, where β-oxidation occurs, are the major source of ROS generation. Fatty acid molecules are broken down to generate acetyl-CoA, which enters the citric acid cycle, and NADH and FADH2 co-enzymes are used in the electron transport chain [53]. Tizioto et al. [54] performed DEG gene analysis between animals that showed high (inefficient) and low (efficient) residual feed intake from the same population used in this study, and indicated the metabolic processes that underlie oxidative stress are a primarily network related to lipid metabolism and energy required for maintenance. These authors also observed the important role of fatty acids in energy metabolism and mitochondrial action on lipid oxidation process similar to that reported in this study.

One of the major transcription factors identified in our study was *PPARGC1A*, which was differentially expressed between the H and L groups for CLA-c9t11 and down-regulated in H group. *PPARGC1A* is a transcriptional coactivator that regulates genes involved in energy metabolism and provides a link between external physiological stimuli and the regulation of mitochondrial biogenesis. *PPARGC1A* regulates global oxidative metabolism by controlling both the induction of mitochondrial metabolism and the removal of its ROS by-products, which could elevate oxidative metabolism and minimize the impact of ROS on cell physiology in muscle [55]. A review by Puigserver and Spiegelman [56] reported that the *PPARGC1A* transcriptional coactivator could be activated or not in response to environmental stimuli or by cellular signals such as cAMP and cytokine pathways, which control energy and nutrient homeostasis. Activated *PPARGC1A* stimulates mitochondrial oxidative metabolism, fiber-type switching in skeletal muscle, and the fasted response in liver [56]. These effects of *PPARGC1A* are the result of gene expression regulation by interacting specifically with other transcription factors such as nuclear hormone receptors, nuclear respiratory factors, and muscle-specific transcription factors.

Another important TF associated with lipid metabolism, *FOXO1*, which regulates the transcriptional cascades of glucose metabolism was differentially expressed in the current study for CLA-c9t11. *FOXO1*, which was down-regulated in the group of animals with high CLA-c9t11 content, is highly expressed in tissues such as pancreas, liver, skeletal muscle and adipose tissue in response to insulin and is associated with metabolic disorders as dyslipidemia (Fig. 5). Dyslipidemia is a metabolic disorder that contributes to the development of atherosclerosis due to the elevation of plasma cholesterol, triglycerides (TGs) or low high-density lipoprotein level [57]. These results are in agreement with a previous study, which indicated that the level of CLA in human diet can influence atherosclerosis in animal models [58].

The functional annotation analysis by IPA® for transcripts associated with FAs with many DEG (i.e. OA and CLA-c9t11) included important TFs, such as *PPARGC1A*, *SREBF1*, *TP53*, *FOXO1* and *MYC* as upstream regulators. In addition, biological processes, which can cause damage to human and animals health, were identified as enriched. Several disorders, diseases, molecular and cellular functions were also identified as associated with OA or CLA-c9t11 FA content in skeletal muscle by transcriptome profiling (Additional files 13 and 18).

Two important genes associated with lipid metabolism, gene expression and cancer were identified as DEGs between animals with high and low CLA-c9t11, specifically, epidermal growth factor receptor (*EGFR,* Fig. 5) and *RNPS* (Fig. 6). *EGFR* is a tyrosine protein kinase, which has been implicated in several biological processes such as cell growth, survival, and migration. The overexpression of *EGFR* was associated with breast, lung and colon cancers [59, 60]. In this study, the *EGFR* was down-regulated in animals with high CLA-c9t11 content, result that agree with the association between CLA-c9t11 content and cancer prevention. *RNPS*, which was up-regulated in the group of animals with high CLA-c9t11 content, can affect several basic functions such as protein synthesis, gene expression, and chromosome stability. It is responsible for pseudouridylation of ribosomal and spliceosomal small nuclear RNAs, ribosomal RNAs processing, and RNA telomerase stabilization [61]. MacDonald [62] and Park et al. [63] reported that association of CLA-c9t11 transcriptome level can regulate cell proliferation and differentiation.

## Conclusions

The effective involvement of OA and CLA-c9t11 in biological processes like transcriptional regulation is still unclear. However, this transcriptome profiling study, which used *Longissimus dorsi* muscle from cattle with extreme values of OA and CLA-c9t11 content, demonstrated that both OA and CLA-c9t11 content had large effects on the expression level of genes related to important biological processes associated with oxidative phosphorylation, cell growth, survival, and migration. It is tempting to speculate that these fatty acids have direct and maybe indirect physiological effects on skeletal muscle, while other fatty acids do not. Additional nutrigenomic and metabolomic studies are necessary to elucidate the metabolic mechanisms by which these fatty acids influence cell growth, survival, and migration, which are interestingly also associated with several human diseases directly related to human dietary intake of fatty acids.

## Methods

### Animals, samples and phenotypes

Three hundred and ninety Nellore steers from the Brazilian Agricultural Research Corporation (EMBRAPA/Brazil) experimental breeding herd between 2009 and 2011 were included in this study; see Cesar et al. [21] for additional details. These steers were sired by 34 unrelated sires that represented most of the main genealogies used in Brazil according to the National Summary of Nellore produced by the Brazilian Association of Zebu Breeders (ABCZ) and National Research Center for Beef Cattle. Animals were raised in feedlots under identical nutrition and handling conditions until slaughter at an average age of 25 months. Samples from LD muscle located between the 12th and 13th ribs were collected in two time-points: at slaughter for RNA sequencing analysis to guarantee the RNA integrity, and 24 h after slaughter for the fatty acid content measurement according to research guidelines for cookery, sensory evaluation and instrumental tenderness measurements of fresh meat [64].

Approximately 100 g samples of LD muscle were lyophilized and ground to a fine powder. Five grams of this ground, lyophilized tissue was used to measure the OA, PA, SA, CLA-c9t11, EPA, DHA and LA content, which were chosen based on their importance in many biological processes and human health [35–40]. The fatty acid content measurement was conducted as described by Hara and Radin [65], except the hexane to propanol ratio was increased to 3:2. Approximately 4 g of LD muscle were lyophilized, ground in liquid nitrogen, mixed with 28 mL of hexane/propanol (3:2 vol/vol) and homogenized for 1 min. Samples were vacuum filtered and 12 mL sodium sulfate (67 mg mL− 1) solution was added and agitated for 30 s. The supernatant was transferred to a tube with 2 g of sodium sulfate and insufflated with nitrogen gas (N_2_), after which the tube was sealed and incubated at room temperature for 30 min. Subsequently, the liquid was transferred to 10 mL test tube, insufflated with N_2_, sealed and kept at -20 °C until dry with N_2_ for methylation. The extracted lipids were hydrolyzed and methylated as described by Christie [66], except that hexane and methyl acetate were used instead of hexane:diethyl ether:formic acid (90:10:1). Around 40 mg of lipids were transferred to a tube containing 2 mL of hexane. Subsequently, 40 μL of methyl acetate were added, the sample agitated, and 40 μL of methylation solution (1.75 mL of methanol/0.4 mL of 5.4 mol/L of sodium metoxyde) were added. This mixture was agitated for 2 min and incubated for 10 min at room temperature. Then 60 μL of finishing solution (1 g of oxalic acid/30 mL of diethyl ether) was added and the mixture was agitated for 30 s, after which 200 mg of calcium chloride was added. The sample was then mixed and incubated at room temperature for 1 h. Samples were centrifuged at 3200 rpm, for 5 min at 5 °C. The supernatant was collected for determination of fatty acid. Fatty acid methyl esters were quantified with a gas chromatograph (ThermoFinnigan, Termo Electron Corp., MA, USA) equipped with a flame ionization detector and a 100 m Supelco SP-2560 (Supelco Inc., PA, USA) fused silica capillary column (100 m, 0.25 mm and 0.2 μ m film thickness). The column oven temperature was held at 70 °C for 4 min, then increased to 170 °C at a rate of 13 °C min − 1, and subsequently increased to 250 °C at a rate of 35 °C min − 1, and held at 250 °C for 5 min. The gas fluxes were 1.8 mL min − 1 for carrier gas (He), 45 mL min − 1 for make-up gas (N_2_), 40 mL min − 1 for hydrogen, and 450 mL min − 1 for synthetic flame gas. One μL sample was analyzed. Injector and detector temperatures were 250 and 300 °C, respectively. The fatty acids were identified by comparison of retention time of methyl esters of the samples with standards of fatty acids butter reference BCR-CRM 164, Anhydrous Milk Fat-Producer (BCR Institute for Materials and Reference Measurements) and also with commercial standard for 37 fatty acids Supelco TM Component FAME Mix (cat 18919, Supelco, Bellefonte, PA). The fatty acid content was quantified by normalizing the area under the curve of methyl esters using Chromquest 4.1 software (Thermo Electron, Italy). The fatty acid content was expressed as a weight percentage (mg/mg). These analyses were performed at the Animal Nutrition and Growth Laboratory at ESALQ, Piracicaba, São Paulo, Brazil.

From a total of 390 animals, we selected 30 animals with either the highest (H) or lowest (L) FA content for each one of the FA chosen (OA, PA, SA, CLA-c9t11, EPA, DHA and LA) to form the groups that were tested for differential expression analysis. Data from some animals were used in more than one analysis, which resulted in a total of 164 animals that were used on this study. The extreme values of FAs were identified based in their adjusted phenotype values, which was estimated via regression as recommended by Naylor et al. [67] and selected for differential gene expression analysis. SAS PROC MIXED (SAS version 9.1) was used to adjust the phenotypic values and run the regression model, in which the contemporary group (animals with the same origin, birth year and slaughter date) and hot carcass weight were fitted as fixed and covariate, respectively. To verify the difference of FA content between the high and low groups, IMF (%) and BFT (mm) a Student’s *t*-test was performed using R package. Because of some samples were used in more than one FA extreme group, the statistical test of means was performed between the high (H) and low (L) groups according to FA by Student’s *t*-test performed in R package. Pairwise correlation analysis was also performed for all FAs. The IMF and BFT measurement were obtained as described by Cesar et al. [21] and Tizioto et al. [68].

### RNA extraction, quality analysis, library preparation and sequencing

Total RNA was extracted from 100 mg of frozen LD muscle that was collected at slaughter using the TRIzol reagent (Life Technologies, Carlsbad, CA). RNA integrity was verified by Bioanalyzer 2100 (Agilent, Santa Clara, CA, USA). Only samples with RIN score > 8 were used. A total of 2 μg of total RNA from each sample was used for library preparation according to the protocol described in the TruSeq RNA Sample Preparation kit v2 guide (Illumina, San Diego, CA). The average insert size of the libraries was estimated using the Agilent Bioanalyzer 2100 (Agilent, Santa Clara, CA, USA) and quantified using quantitative PCR with the KAPA Library Quantification kit (KAPA Biosystems, Foster City, CA, USA). Quantified, samples were diluted and pooled (three pools of six samples each). Three lanes of a sequencing flowcell, using the TruSeq PE Cluster kit v3-cBot-HS kit (Illumina, San Diego, CA, USA), were clustered and sequenced using HiSeq2500 ultra-high-throughput sequencing system (Illumina, San Diego, CA, USA) with the TruSeq SBS Kit v3-HS (200 cycles), according to manufacturer instructions. The sequencing analyses were performed at the Genomics Center at ESALQ, Piracicaba, São Paulo, Brazil.

### Quality control and read alignment

Sequencing adaptors and low-complexity reads were removed in an initial data filtering step. Quality control and reads statistics were estimated with FASTQC version 0.10.1 software [http://www.bioinformatics.bbsrc.ac.uk/projects/fastqc/]. Tophat v. 2.0.11 software [69] with Bowtie2 (version 2.2.3) [70] was used to map reads to the UMD3.1 *Bos taurus* reference assembly available at Ensembl [http://www.ensembl.org/Bos_taurus/Info/Index/]. The abundance (read counts) of mRNAs for all annotated genes was calculated using HTSeq version 0.6.1 software [http://www-huber.embl.de/users/anders/HTSeq/] [71]. Only sequence reads that uniquely mapped to known chromosomes (excluding reads mapped to unassigned contigs) were used in further analyses.

### Identification of differential expressed genes and pathway analysis

Differentially expressed genes were identified using the DESeq2 statistical package available at Bioconductor open source software for bioinformatics [72], using multi-factor design. Prior to statistical analysis, read count data was filtered as follows: i) transcripts with zero counts were removed (unexpressed); ii) genes with less than 1 read per sample on average were removed (very lowly expressed); iii) genes that were not present in at least three samples were removed (rarely expressed). Transcript expression level was fit as a negative binomial distribution. Age (covariate) and contemporary group (animals with the same origin, birth year and slaughter date; class effect) were fit as factors in the multi-factor model. Exploratory diagnostic plots were generated to check the dispersion estimates. The cut-off approach performed to identify the DEGs was Benjamini-Hochberg [15] methodology was used to control false discovery rate (FDR) at 10%.

Enrichment analysis of gene ontology terms was completed with the Database for Annotation, Visualization and Integrated Discovery (DAVID) v6.7 tool [73] using the list of genes that presented FDR < 10%. The functional analysis of statistically significant gene expression changes (FDR 10%) between the groups was performed with QIAGEN’s Ingenuity® Pathways Analysis (IPA®, QIAGEN Redwood City, www.qiagen.com/ingenuity), which uses the Fisher’s exact test to calculate a *p*-value for each biological function assigned to the experimental data. These analyses were performed just for OA and CLA-c9t11 because they presented higher impact on gene expression level.

### Quantitative real-time PCR analysis

Total RNAs of three samples of each group (H and L) and nine DE genes were randomly selected for quantitative PCR analysis. The High Capacity cDNA Reverse Transcription Kit (Life Technologies, Grand Island, NY) was performed in reverse transcription (RT) as manufacturer’s procedure. The RT reaction contained 150 ng of total RNA, 1 μL of 10× RT buffer, 0.5 μl of 25× dNTP mixture, 1 μL of 10× random reverse primers, 1 μL of 10× gene-specific reverse primers (1 μM) and 0.5 μLof MultiScribe RT (50 U/μl). The reactions of 10 μL were incubated in thermocycler for 10 min at 25 °C, 2 h at 37 °C and then held at 4 °C. All samples were treated with Ambion TURBO DNA-free® DNase (Life Technologies, Grand Island, NY) to remove contaminating DNA from RNA preparations. Quantitative real-time PCR was performed on an Applied Biosystems QuantStudio™ 12 K Flex System (Thermo Fisher Scientific, Waltham, MA) using the TaqMan OpenArray gene expression plates. The TaqMan assays of nine DE genes selected (*NDUFV1*, *NDUFS6*, *NDUFB7*, *COX7A1*, *CYP4B1*, *JAM2*, *PLIN5*, *COA5* and *NDUFS8*) for this study was designed and performed by manufacturer (http://www.thermofisher.com/order/genome-database). The PCR reaction (1.2 μLof sample plus 3.8 μL of PCR mix) was prepared for all samples and placed into 384-well sample plate. The PCR reactions were dispensed using the Accufill System onto the corresponding OpenArray plate. The PCR condition was performed as manufacturer’s procedure. The comparative Ct method for quantification was performed to quantify the relative expression of specific genes by QuantStudio 12 K Flex Software (Thermo Fisher Scientific, Waltham, MA) according to Pfaffl [74]. Eukaryotic Translation Elongation Factor 1 Alpha 2 (*EEF1A2* - Bt03229236_mH) and Beta-2-Microglobulin (*B2M* - Bt03251628_m1) were selected as reference genes to normalize gene expression. Analysis of covariance (ANCOVA) [75] using the same fixed effect and covariate used in RNA-Seq analysis was performed to test the significance (*p* < 0.10) of FA content (group effect) on gene expression level of the DE genes selected randomly for this study.

### Data availability

The dataset supporting the conclusions of this article is available in the in the European Nucleotide Archive (ENA) repository (EMBL-EBI), under accession PRJEB13188 [http://www.ebi.ac.uk/ena/data/view/PRJEB13188].

## Additional files

Additional file 1: Phenotypic and statistical analysis description for two groups of extreme values of fatty acids content in Longissimus dorsi muscle of Nellore steers. (XLSX 43 kb)
Additional file 2: Test of means (*t*-test) between groups with high and low fatty acid content in Longissimus dorsi muscle in Nellore steers. (XLSX 44 kb)
Additional file 3: Summary of total reads before and after filtering, percentage of mapped reads against Bos taurus UMD3.1 reference genome and, information of each group according to specifically fatty acid content from Longissimus dorsi muscle of Nellore steers. (XLSX 53 kb)
Additional file 4: Normalized gene expression level and statistical results of differentially expressed genes analysis (FDR 10%) between High and Low groups based on linoleic acid (LA) content from Longissimus dorsi muscle of Nellore steers. (XLSX 15462 kb)
Additional file 5: Normalized gene expression level and statistical results of differentially expressed genes analysis (FDR 10%) between High and Low groups based on stearic acid (SA) content from Longissimus dorsi muscle of Nellore steers. (XLSX 15406 kb)
Additional file 6: Normalized gene expression level and statistical results of differentially expressed genes analysis (FDR 10%) between High and Low groups based on eicosapentaenoic acid (EPA) content from Longissimus dorsi muscle of Nellore steers. (XLSX 15447 kb)
Additional file 7: Normalized gene expression level and statistical results of differentially expressed genes analysis (FDR 10%) between High and Low groups based on docosahexaenoic acid (DHA) content from Longissimus dorsi muscle of Nellore steers. (XLSX 13199 kb)
Additional file 8: Normalized gene expression level and statistical results of differentially expressed genes analysis (FDR 10%) between High and Low groups based on palmitic acid (PA) content from Longissimus dorsi muscle of Nellore steers. (XLSX 15040 kb)
Additional file 9: Normalized gene expression level and statistical results of differentially expressed genes analysis (FDR 10%) between High and Low groups based on oleic acid (OA) content from Longissimus dorsi muscle of Nellore steers. (XLSX 13046 kb)
Additional file 10: Normalized gene expression level and statistical results of differentially expressed genes analysis (FDR 10%) between High and Low groups based on conjugated linoleic acid cis9 trans11 (CLA-c9t11) content from Longissimus dorsi muscle of Nellore steers. (XLSX 15002 kb)
Additional file 11: Volcano plot of log2 fold-change (x-axis) versus − log10 FDR-corrected *p*-value in RNA-Seq data for (A) palmitic acid (PA) content, (B) docosapentaenoic acid (DHA) content, (C) eicosapentaenoic (EPA) content, (D) linoleic acid (LA) content and, (E) stearic acid (SA) content (F) conjugated linoleic acid (CLA) content. (DOCX 887 kb)
Additional file 12: Functional enrichment and significant category (BH-adj <10%) are shown from differentially expressed genes (FDR 10%) between High and Low groups based on oleic acid (OA) content from Longissimus dorsi muscle of Nellore steers. (DOCX 127 kb)
Additional file 13: Enriched canonical pathways of the differentially expressed genes between High and Low groups based on oleic acid content from Longissimus dorsi muscle of Nellore steers using Ingenuity Pathway Analysis (IPA). (XLS 83 kb)
Additional file 14: IPA network analysis of differentially expressed genes analysis (FDR 10%) between High and Low groups based on oleic acid content from Longissimus dorsi muscle of Nellore steers. (XLSX 57 kb)
Additional file 15: Uspstrem regulators analysis from DE genes list. (XLS 27 kb)
Additional file 16: Biological functions identified by IPA from differentially expressed genes list (FDR 10%) between high and low groups based on oleic acid content in Longissimus dorsi muscle of Nellore steers. (XLSX 57 kb)
Additional file 17: Functional enrichment and significant category (BH-adj <10%) are shown from differentially expressed genes (FDR 10%) between High and Low groups based on conjugated linoleic acid (CLA-c9t11) content from Longissimus dorsi muscle of Nellore steers. (DOCX 95 kb)
Additional file 18: Summary of functional enrichment analysis from a list of differentially expressed genes between High and Low groups based on conjugated linoleic acid cis9 trans11 content from Longissimus dorsi muscle of Nellore steers using Ingenuity Pathway Analysis. (IPA) (XLSX 12 kb)
Additional file 19: Cytokines and transcription regulator identified as upstream regulators by Ingenuity Pathway Analysis (IPA) as inhibited or activated from a list of differential expressed genes between High and Low groups based on conjugated linoleic acid cis9 trans11 (CLA-c9t11) content from Longissimus dorsi muscle of Nellore steers. (DOCX 95 kb)
Additional file 20: qPCR results of nine differentially expressed genes by random selection from DataAssist software. (XLSX 49 kb)
Additional file 21: qPCR validation of RNA-seq data on a random selection of nine differentially expressed genes. (XLSX 39 kb)

## Abbreviations

ABCZ: Brazilian Association of Zebu Breeders
BFT: Backfat thickness
BH: Benjamin-Hochberg
CLA: Conjugated linoleic acid
CLA-c9t11: Conjugated linoleic acid cis 9, trans 11
DAVID: Database for annotation, visualization and integrated discovery
DEG: Differentially expressed gene
DHA: Docosahexaenoic acid
EPA: Eicosapentaenoic acid
FA: Fatty acid
FDR: False discovery rate
GCP: Global canonical pathways
GFA: Global functional analysis
H: Group high
HDL: High-density lipoprotein
IMF: Intramuscular fat
IPA: Ingenuity pathways analysis
KEGG: Kyoto encyclopedia of genes and genomes
L: Group low
LA: Linoleic acid
LD: *Longissimus dorsi*
LDL: Low-density lipoprotein
MUFA: Monounsaturated fatty acidOA: Oleic acid
OEA: Oleoylethanolamide
PA: Palmitic acid
PCR: Polymerase chain reaction
PUFA: Polyunsaturated fatty acid
qRT-PCR: Quantitative real-time polymerase chain reaction
ROS: Reactive oxygen species
SA: Stearic acid
SFA: Saturated fatty acid
TF: Transcription factor
VLDL: Very low-density lipoprotein
