# Supplementary material for: Differences in the skeletal muscle transcriptome profile associated with extreme values of fatty acids content
Source: BMC Genomics. 2016 Nov 22;17:961. doi: 10.1186/s12864-016-3306-x (PMC5120530; doi:10.1186/s12864-016-3306-x)
Supplement: Additional file 11: — Volcano plot of log2 fold-change (x-axis) versus − log10 FDR-corrected p-value in RNA-Seq data for (A) palmitic acid (PA) content, (B) docosapentaenoic acid (DHA) content, (C) eicosapentaenoic (EPA) content, (D) linoleic acid (LA) content and, (E) stearic acid (SA) content (F) conjugated linoleic acid (CLA) content. (DOCX 887 kb) [file 12864_2016_3306_MOESM11_ESM.docx]

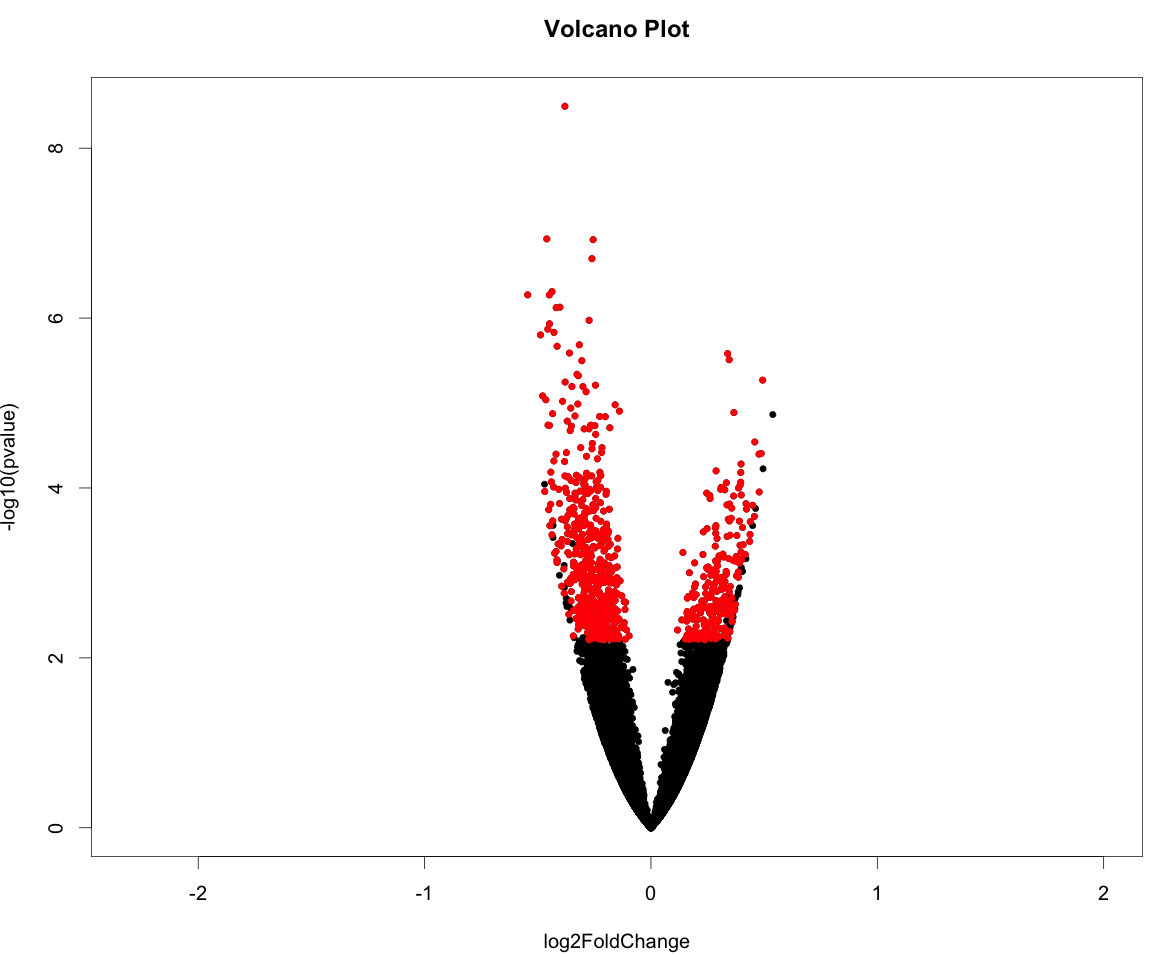


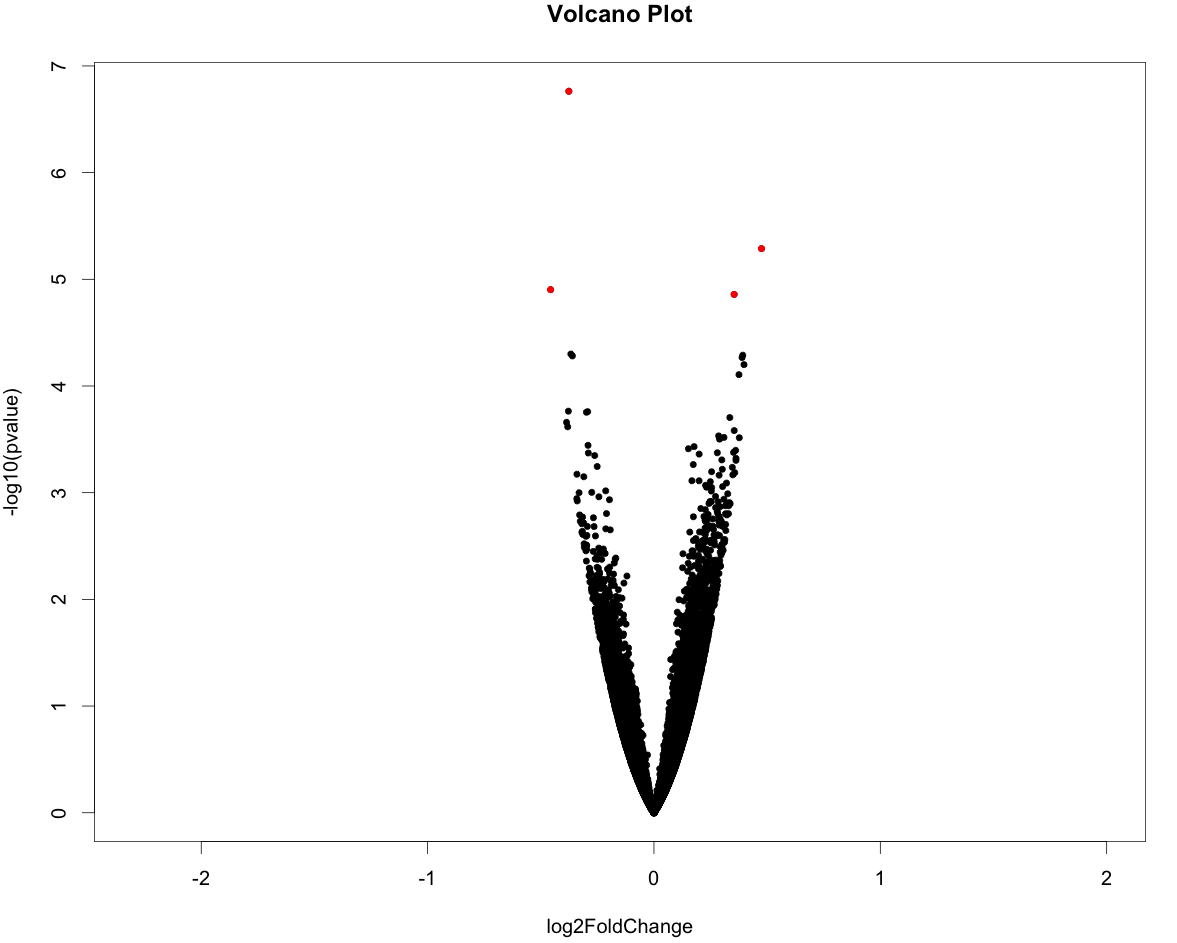

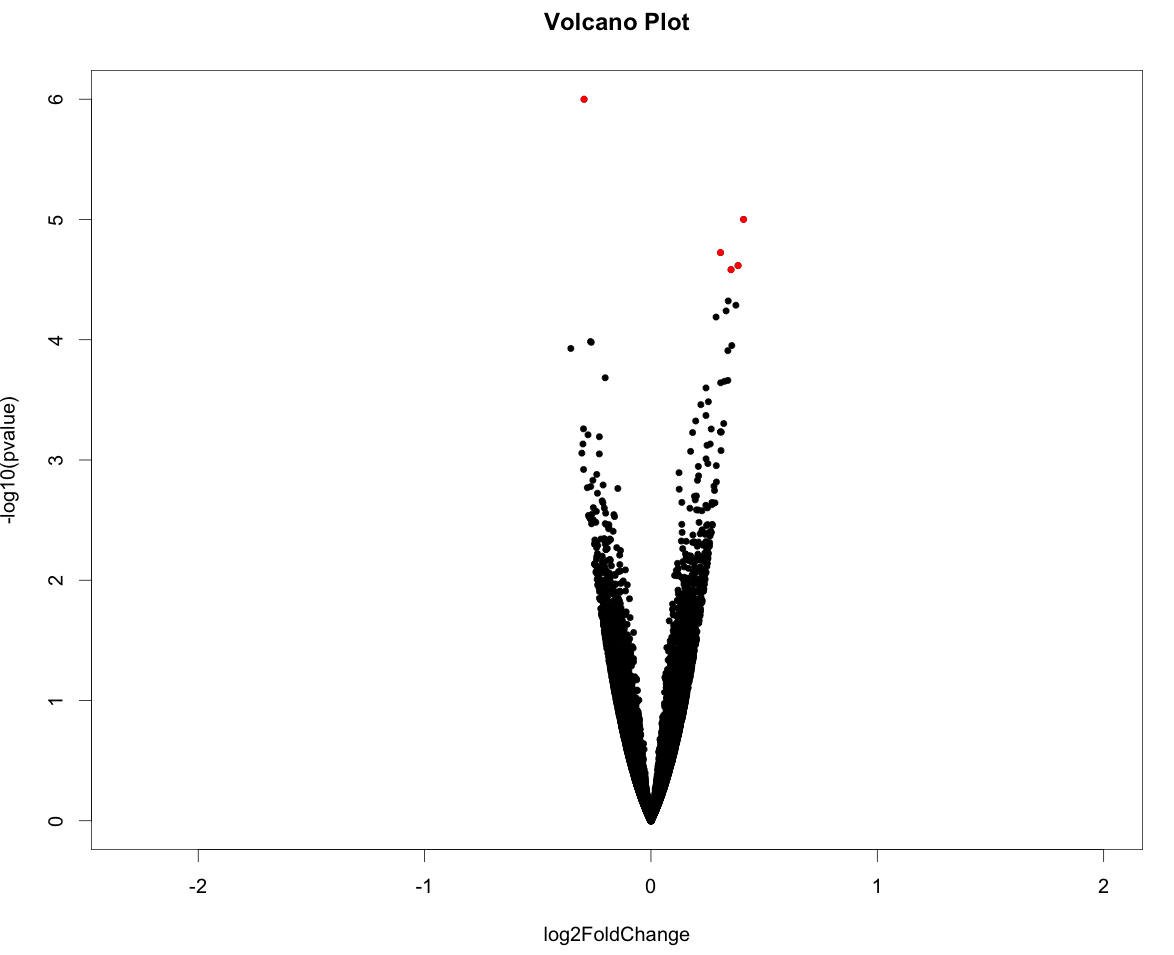

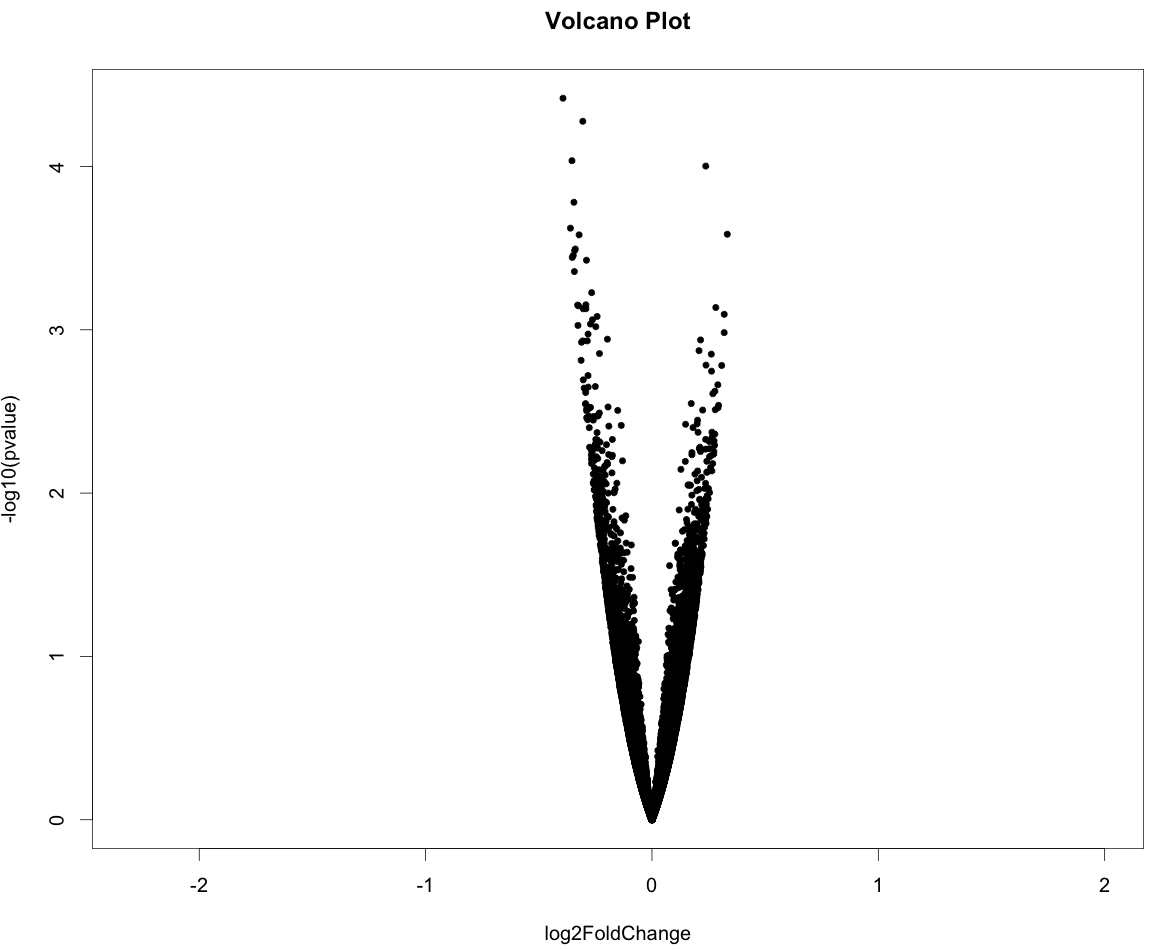

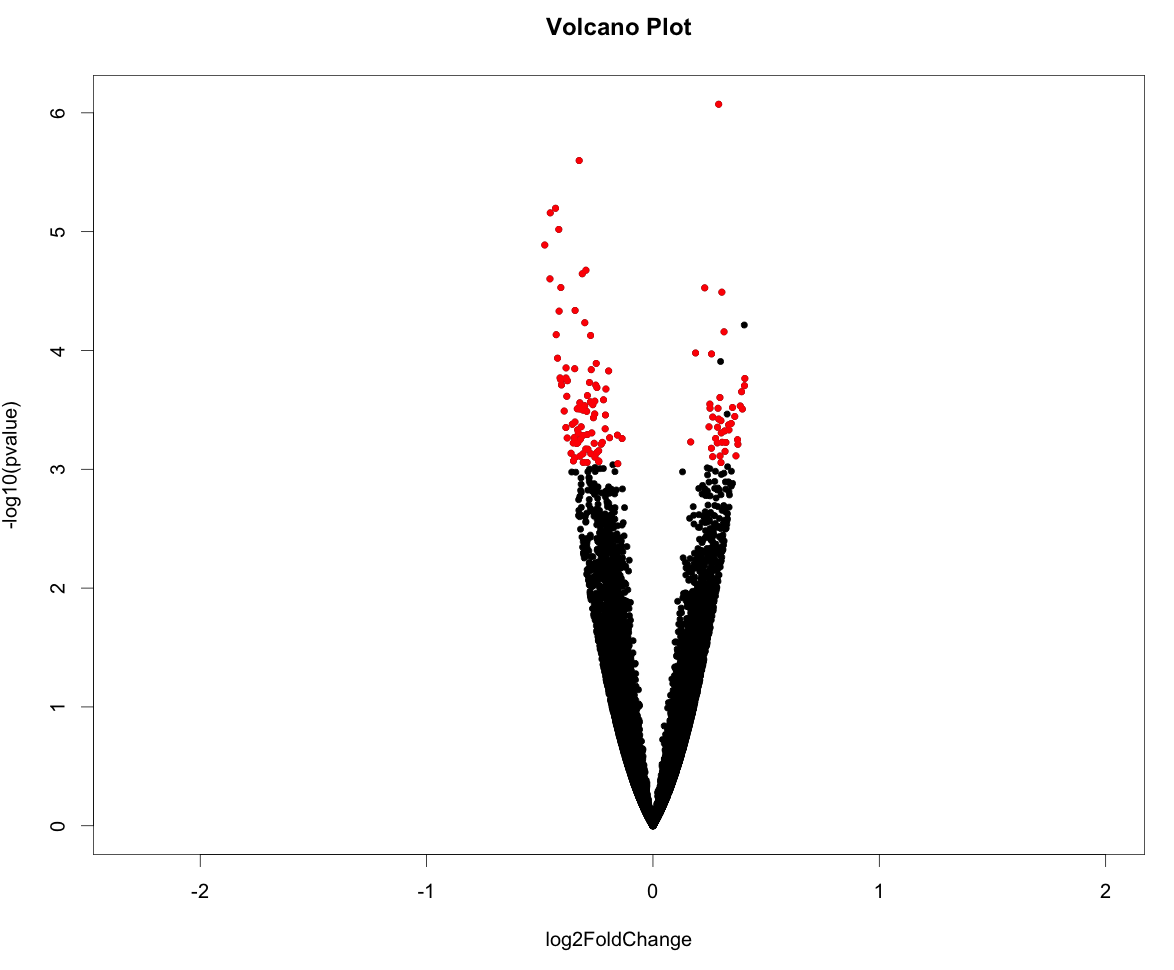

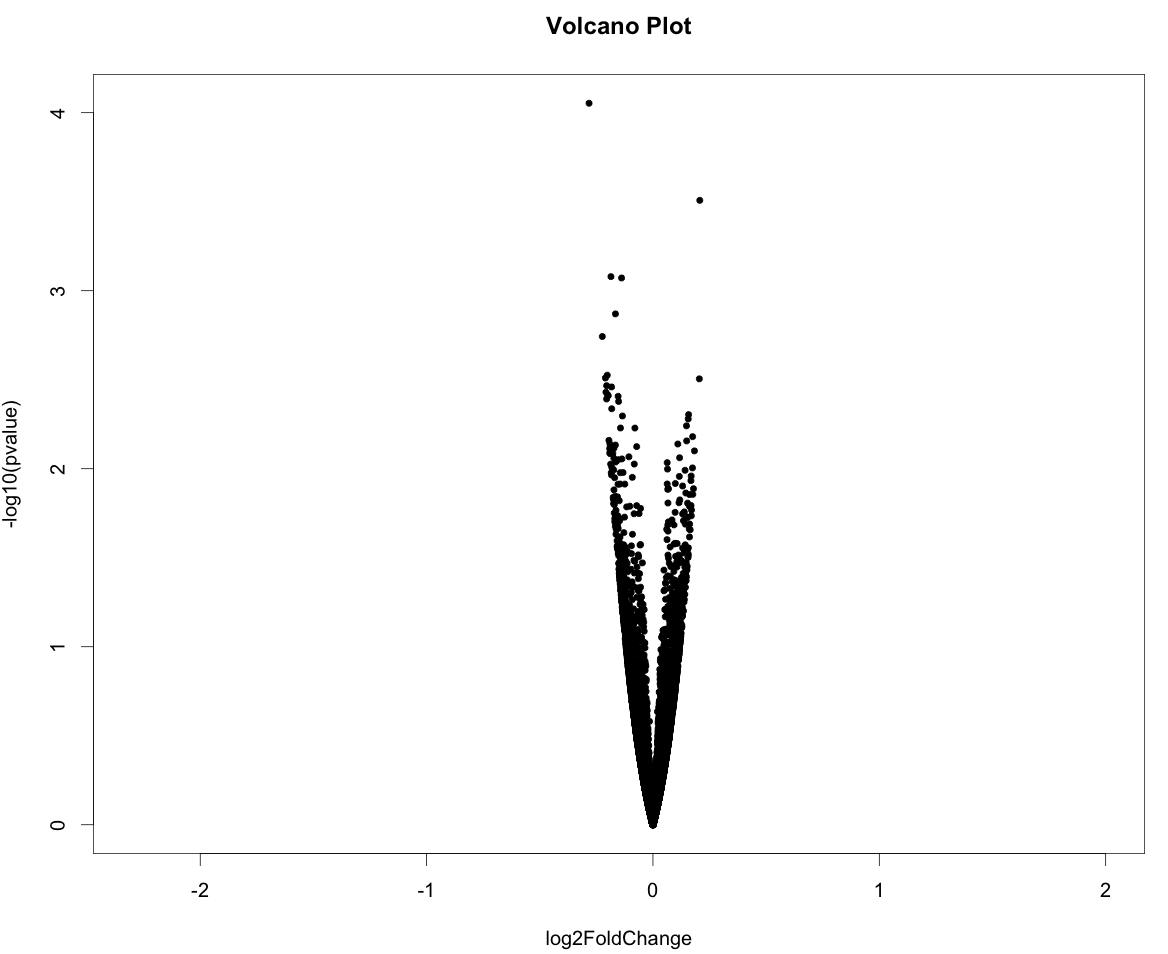


A

B

C

D

E

F

Additional File 11. Volcano plot of log2 fold-change (x-axis) versus −log10 FDR-corrected p-value in RNA-Seq data for (A) palmitic acid (PA) content, (B) docosapentaenoic acid (DHA) content, (C) eicosapentaenoic (EPA) content, (D) linoleic acid (LA) content and, (E) stearic acid (SA) content (F) conjugated linoleic acid (CLA) content.
