# Supplementary material for: Differences in the skeletal muscle transcriptome profile associated with extreme values of fatty acids content
Source: BMC Genomics. 2016 Nov 22;17:961. doi: 10.1186/s12864-016-3306-x (PMC5120530; doi:10.1186/s12864-016-3306-x)
Supplement: Additional file 12: — Functional enrichment and significant category (BH-adj <10%) are shown from differentially expressed genes (FDR 10%) between High and Low groups based on oleic acid (OA) content from Longissimus dorsi muscle of Nellore steers. (DOCX 127 kb) [file 12864_2016_3306_MOESM12_ESM.docx]

Additional File 12. Functional enrichment and significant category (BH-adj <10%) are shown from differentially expressed genes (FDR 10%) between High and Low groups based on oleic acid (OA) content from *Longissimus dorsi* muscle of Nellore steers.

| **Category** | **Term** | **Count^1^** | **P-Value** | **BH-adj**^2^ |
| --- | --- | --- | --- | --- |
| GOTERM_CC_FAT | mitochondrion | 136 | 7.70e-36 | 2.60e-33 |
| GOTERM_CC_FAT | mitochondrial part | 83 | 1.40e-25 | 2.30e-23 |
| SP_PIR_KEYWORDS | mitochondrion | 107 | 1.50e-23 | 4.80e-21 |
| GOTERM_CC_FAT | mitochondrial envelope | 60 | 2.30e-17 | 2.60e-15 |
| GOTERM_CC_FAT | mitochondrial inner membrane | 53 | 7.30e-17 | 9.30e-15 |
| GOTERM_CC_FAT | organelle inner membrane | 53 | 1.90e-16 | 1.50e-14 |
| GOTERM_CC_FAT | mitochondrial membrane | 57 | 3.30e-16 | 1.90e-14 |
| GOTERM_CC_FAT | ribonucleoprotein complex | 63 | 4.20e-15 | 2.00e-13 |
| GOTERM_CC_FAT | organelle envelope | 65 | 1.60e-14 | 6.60e-13 |
| GOTERM_CC_FAT | envelope | 65 | 1.80e-14 | 6.70e-13 |
| SP_PIR_KEYWORDS | ribonucleoprotein | 51 | 2.10e-14 | 2.20e-12 |
| SP_PIR_KEYWORDS | mitochondrion inner membrane | 42 | 1.60e-14 | 2.50e-12 |
| SP_PIR_KEYWORDS | ribosomal protein | 44 | 6.20e-14 | 5.00e-12 |
| GOTERM_CC_FAT | ribosome | 44 | 1.90e-13 | 6.20e-12 |
| GOTERM_CC_FAT | respiratory chain | 25 | 2.20e-13 | 6.60e-12 |
| GOTERM_BP_FAT | electron transport chain | 29 | 1.80e-14 | 2.80e-11 |
| SP_PIR_KEYWORDS | transit peptide | 61 | 2.70e-12 | 1.50e-10 |
| SP_PIR_KEYWORDS | respiratory chain | 27 | 2.60e-12 | 1.70e-10 |
| SP_PIR_KEYWORDS | electron transport | 29 | 5.80e-12 | 2.70e-10 |
| KEGG_PATHWAY | Huntington's disease | 38 | 3.90e-12 | 3.00e-10 |
| KEGG_PATHWAY | Oxidative phosphorylation | 33 | 3.70e-12 | 5.80e-10 |
| GOTERM_MF_FAT | structural constituent of ribosome | 37 | 1.20e-12 | 6.50e-10 |
| GOTERM_BP_FAT | translation | 50 | 1.80e-12 | 1.40e-09 |
| KEGG_PATHWAY | Parkinson's disease | 30 | 2.90e-10 | 1.50e-08 |
| GOTERM_CC_FAT | organelle membrane | 71 | 5.90e-10 | 1.60e-08 |
| GOTERM_CC_FAT | mitochondrial lumen | 27 | 2.30e-09 | 6.00e-08 |
| GOTERM_CC_FAT | mitochondrial matrix | 27 | 2.30e-09 | 6.00e-08 |
| UP_SEQ_FEATURE | transit peptide:Mitochondrion | 61 | 9.20e-11 | 9.40e-08 |
| KEGG_PATHWAY | Alzheimer's disease | 32 | 3.20e-09 | 1.20e-07 |
| GOTERM_CC_FAT | ribosomal subunit | 17 | 2.80e-08 | 6.60e-07 |
| GOTERM_BP_FAT | generation of precursor metabolites and energy | 37 | 2.70e-09 | 1.40e-06 |
| GOTERM_CC_FAT | membrane-enclosed lumen | 70 | 1.30e-07 | 3.00e-06 |
| GOTERM_CC_FAT | mitochondrial ribosome | 11 | 2.10e-07 | 4.50e-06 |
| GOTERM_CC_FAT | organellar ribosome | 11 | 2.10e-07 | 4.50e-06 |
| GOTERM_CC_FAT | intracellular organelle lumen | 67 | 2.90e-07 | 5.80e-06 |
| GOTERM_CC_FAT | organelle lumen | 67 | 3.10e-07 | 5.80e-06 |
| GOTERM_BP_FAT | oxidation reduction | 62 | 2.70e-08 | 1.10e-05 |
| SP_PIR_KEYWORDS | acetylation | 139 | 2.70e-07 | 1.10e-05 |
| GOTERM_CC_FAT | mitochondrial large ribosomal subunit | 8 | 3.70e-06 | 6.60e-05 |
| GOTERM_CC_FAT | organellar large ribosomal subunit | 8 | 3.70e-06 | 6.60e-05 |
| GOTERM_CC_FAT | large ribosomal subunit | 10 | 6.20e-06 | 1.00e-04 |
| GOTERM_MF_FAT | NADH dehydrogenase activity | 11 | 5.00e-07 | 1.40e-04 |
| GOTERM_CC_FAT | non-membrane-bounded organelle | 104 | 1.40e-05 | 2.30e-04 |
| GOTERM_CC_FAT | intracellular non-membrane-bounded organelle | 104 | 1.40e-05 | 2.30e-04 |
| GOTERM_MF_FAT | structural molecule activity | 45 | 1.60e-06 | 3.00e-04 |
| GOTERM_MF_FAT | NADH dehydrogenase (quinone) activity | 10 | 3.60e-06 | 5.00e-04 |
| GOTERM_MF_FAT | NADH dehydrogenase (ubiquinone) activity | 10 | 3.60e-06 | 5.00e-04 |
| GOTERM_MF_FAT | oxidoreductase activity, acting on NADH or NADPH | 13 | 5.30e-06 | 6.00e-04 |
| SP_PIR_KEYWORDS | oxidoreductase | 52 | 2.30e-05 | 8.30e-04 |
| GOTERM_MF_FAT | oxidoreductase activity, acting on NADH or NADPH, quinone or similar compound as acceptor | 10 | 1.10e-05 | 9.80e-04 |
| GOTERM_CC_FAT | mitochondrial membrane part | 12 | 1.30e-04 | 2.10e-03 |
| GOTERM_MF_FAT | monovalent inorganic cation transmembrane transporter activity | 16 | 3.40e-05 | 2.70e-03 |
| GOTERM_MF_FAT | inorganic cation transmembrane transporter activity | 18 | 5.20e-05 | 3.60e-03 |
| GOTERM_BP_FAT | cellular protein catabolic process | 33 | 1.30e-05 | 4.00e-03 |
| GOTERM_MF_FAT | hydrogen ion transmembrane transporter activity | 15 | 7.80e-05 | 4.80e-03 |
| GOTERM_BP_FAT | protein catabolic process | 34 | 2.40e-05 | 6.40e-03 |
| GOTERM_BP_FAT | proteolysis involved in cellular protein catabolic process | 32 | 3.00e-05 | 6.80e-03 |
| GOTERM_CC_FAT | mitochondrial respiratory chain | 7 | 6.80e-04 | 9.90e-03 |
| GOTERM_MF_FAT | RNA binding | 36 | 2.40e-04 | 1.20e-02 |
| GOTERM_MF_FAT | electron carrier activity | 20 | 2.40e-04 | 1.30e-02 |
| GOTERM_BP_FAT | ncRNA metabolic process | 21 | 7.20e-05 | 1.40e-02 |
| KEGG_PATHWAY | Ribosome | 15 | 5.00e-04 | 1.60e-02 |
| GOTERM_BP_FAT | RNA processing | 32 | 1.20e-04 | 1.90e-02 |
| GOTERM_BP_FAT | cellular macromolecule catabolic process | 34 | 1.20e-04 | 2.10e-02 |
| GOTERM_BP_FAT | ncRNA processing | 17 | 1.90e-04 | 2.70e-02 |
| KEGG_PATHWAY | Proteasome | 10 | 1.10e-03 | 2.90e-02 |
| GOTERM_CC_FAT | proteasome complex | 10 | 2.10e-03 | 2.90e-02 |
| GOTERM_BP_FAT | macromolecule catabolic process | 36 | 2.50e-04 | 3.30e-02 |
| GOTERM_BP_FAT | modification-dependent protein catabolic process | 27 | 5.20e-04 | 6.20e-02 |
| GOTERM_BP_FAT | modification-dependent macromolecule catabolic process | 27 | 5.20e-04 | 6.20e-02 |
| GOTERM_CC_FAT | small ribosomal subunit | 7 | 4.90e-03 | 6.40e-02 |
| GOTERM_CC_FAT | cytosol | 29 | 5.90e-03 | 7.40e-02 |
| GOTERM_CC_FAT | DNA-directed RNA polymerase complex | 5 | 6.30e-03 | 7.50e-02 |
| GOTERM_CC_FAT | RNA polymerase complex | 5 | 6.30e-03 | 7.50e-02 |
| GOTERM_CC_FAT | nuclear DNA-directed RNA polymerase complex | 5 | 6.30e-03 | 7.50e-02 |

^1^ Number of differentially expressed genes involved in the term

^2^ P-value adjusted for multiple tests by Benjamin and Hochberg (1995)
