# Supplementary material for: Differences in the skeletal muscle transcriptome profile associated with extreme values of fatty acids content
Source: BMC Genomics. 2016 Nov 22;17:961. doi: 10.1186/s12864-016-3306-x (PMC5120530; doi:10.1186/s12864-016-3306-x)
Supplement: Additional file 17: — Functional enrichment and significant category (BH-adj <10%) are shown from differentially expressed genes (FDR 10%) between High and Low groups based on conjugated linoleic acid (CLA-c9t11) content from Longissimus dorsi muscle of Nellore steers. (DOCX 95 kb) [file 12864_2016_3306_MOESM17_ESM.docx]

Additional File 17. Functional enrichment and significant category (BH-adj <10%) are shown from differentially expressed genes (FDR 10%) between High and Low groups based on conjugated linoleic acid (CLA-c9t11) content from *Longissimus dorsi* muscle of Nellore steers.

| **Category** | **Term** | **Count^1^** | **P-Value** | **BH-adj^2^** |
| --- | --- | --- | --- | --- |
| GOTERM_MF_FAT | nucleotide binding | 99 | 3.30e-06 | 1.70e-03 |
| GOTERM_MF_FAT | purine nucleotide binding | 85 | 1.20e-05 | 3.00e-03 |
| GOTERM_MF_FAT | purine ribonucleotide binding | 80 | 4.30e-05 | 7.40e-03 |
| GOTERM_MF_FAT | ribonucleotide binding | 80 | 4.30e-05 | 7.40e-03 |
| KEGG_PATHWAY | Ribosome | 14 | 9.40e-05 | 1.30e-02 |
| GOTERM_MF_FAT | adenyl nucleotide binding | 68 | 1.00e-04 | 1.30e-02 |
| GOTERM_MF_FAT | purine nucleoside binding | 68 | 1.30e-04 | 1.30e-02 |
| GOTERM_MF_FAT | nucleoside binding | 68 | 1.60e-04 | 1.30e-02 |
| GOTERM_BP_FAT | protein localization | 39 | 9.80e-06 | 1.60e-02 |
| GOTERM_BP_FAT | establishment of protein localization | 35 | 3.80e-05 | 2.10e-02 |
| GOTERM_MF_FAT | ATP binding | 63 | 3.00e-04 | 2.20e-02 |
| GOTERM_MF_FAT | structural constituent of ribosome | 18 | 4.10e-04 | 2.30e-02 |
| GOTERM_MF_FAT | adenyl ribonucleotide binding | 63 | 3.80e-04 | 2.40e-02 |
| GOTERM_BP_FAT | protein transport | 35 | 3.60e-05 | 2.90e-02 |
| SP_PIR_KEYWORDS | ribosomal protein | 20 | 8.20e-04 | 5.50e-02 |
| GOTERM_CC_FAT | ribosome | 21 | 4.70e-04 | 7.00e-02 |
| GOTERM_CC_FAT | ribosome | 21 | 4.70e-04 | 7.00e-02 |

^1^ Number of differentially expressed genes involved in the term

^2^ P-value adjusted for multiple tests by Benjamin and Hochberg (1995)
