# Supplementary material for: Differences in the skeletal muscle transcriptome profile associated with extreme values of fatty acids content
Source: BMC Genomics. 2016 Nov 22;17:961. doi: 10.1186/s12864-016-3306-x (PMC5120530; doi:10.1186/s12864-016-3306-x)
Supplement: Additional file 19: — Cytokines and transcription regulator identified as upstream regulators by Ingenuity Pathway Analysis (IPA) as inhibited or activated from a list of differential expressed genes between High and Low groups based on conjugated linoleic acid cis9 trans11 (CLA-c9t11) content from Longissimus dorsi muscle of Nellore steers. (DOCX 95 kb) [file 12864_2016_3306_MOESM19_ESM.docx]

Additional File 19. Cytokines and transcription regulator identified as upstream regulators by Ingenuity Pathway Analysis (IPA) as inhibited or activated from a list of differential expressed genes between High and Low groups based on conjugated linoleic acid cis9 trans11 (CLA-c9t11) content from *Longissimus dorsi* muscle of Nellore steers.

| **Upstream regulators** | **Molecule Type** | **Predicted Activation** | **Activation z-score** |
| --- | --- | --- | --- |
| TRIM24 | transcription regulator | inhibited | -3.592 |
| IL1RN | cytokine | inhibited | -3.464 |
| NKX2-3 | transcription regulator | inhibited | -2.760 |
| FOXC2 | transcription regulator | inhibited | -2.236 |
| IRF4 | transcription regulator | inhibited | -2.164 |
| IRF2 | transcription regulator | inhibited | -2.156 |
| SOX2 | transcription regulator | inhibited | -2.043 |
| ZBTB16 | transcription regulator | inhibited | -2.000 |
| GATA3 | transcription regulator | inhibited | -2.000 |
| PSMD10 | transcription regulator | inhibited | -2.000 |
| IFNK | cytokine | activated | 2.000 |
| IL1A | cytokine | activated | 2.000 |
| ECSIT | transcription regulator | activated | 2.000 |
| IL5 | cytokine | activated | 2.010 |
| MYC | transcription regulator | activated | 2.036 |
| IL17A | cytokine | activated | 2.049 |
| NFKB1A | transcription regulator | activated | 2.087 |
| JUN | transcription regulator | activated | 2.110 |
| FOXO1 | transcription regulator | activated | 2.126 |
| HMGB1 | transcription regulator | activated | 2.150 |
| IFNA21 | cytokine | activated | 2.193 |
| CCL5 | cytokine | activated | 2.194 |
| IFNA10 | cytokine | activated | 2.197 |
| IFNA5 | cytokine | activated | 2.197 |
| IFNA7 | cytokine | activated | 2.197 |
| IFNA4 | cytokine | activated | 2.197 |
| IFNA16 | cytokine | activated | 2.197 |
| IFNA14 | cytokine | activated | 2.214 |
| IRF16 | transcription regulator | activated | 2.219 |
| MSC | transcription regulator | activated | 2.236 |
| SREBF1 | transcription regulator | activated | 2.291 |
| IL21 | cytokine | activated | 2.333 |
| IFNA6 | cytokine | activated | 2.369 |
| MYCN | transcription regulator | activated | 2.399 |
| TNFSF12 | cytokine | activated | 2.584 |
| IFNA2TNFSF10 | cytokine | activated | 2.630 |
| IL2TNF | cytokine | activated | 2.653 |
| NFATC2 | transcription regulator | activated | 2.720 |
| TP53 | transcription regulator | activated | 2.836 |
| IRF5 | transcription regulator | activated | 2.929 |
| STAT2 | transcription regulator | activated | 2.931 |
| IRF1 | transcription regulator | activated | 2.939 |
| PRL | cytokine | activated | 2.971 |
| RELA | transcription regulator | activated | 3.039 |
| IFNA8 | cytokine | activated | 3.205 |
| IFNA1/IFNA13 | cytokine | activated | 3.234 |
| IL1B | cytokine | activated | 3.236 |
| STAT1 | transcription regulator | activated | 3.495 |
| IFNL1 | cytokine | activated | 3.533 |
| IRF3 | transcription regulator | activated | 3.610 |
| BRCA1 | transcription regulator | activated | 3.697 |
| IL18 | cytokine | activated | 3.995 |
| IFNG | cytokine | activated | 4.050 |
| IRF7 | transcription regulator | activated | 4.549 |
| IFNB1 | cytokine | activated | 5.255 |
